# Supplementary material for: Transitions in lineage specification and gene regulatory networks in hematopoietic stem/progenitor cells over human development
Source: Cell Rep. 2021 Sep 14;36(11):109698. doi: 10.1016/j.celrep.2021.109698 (PMC8456780; doi:10.1016/j.celrep.2021.109698)
Supplement: Document S1. Figures S1–S5 [file mmc1.pdf]

Cell Reports, Volume *36*

## Supplemental information

# Transitions in lineage specification and gene regulatory networks in hematopoietic stem/progenitor cells over human development

**Anindita Roy, Guanlin Wang, Deena Iskander, Sorcha O'Byrne, Natalina Elliott, Jennifer O'Sullivan, Gemma Buck, Elisabeth F. Heuston, Wei Xiong Wen, Alba Rodriguez Meira, Peng Hua, Anastasios Karadimitris, Adam J. Mead, David M. Bodine, Irene Roberts, Bethan Psaila, and Supat Thongjuea**

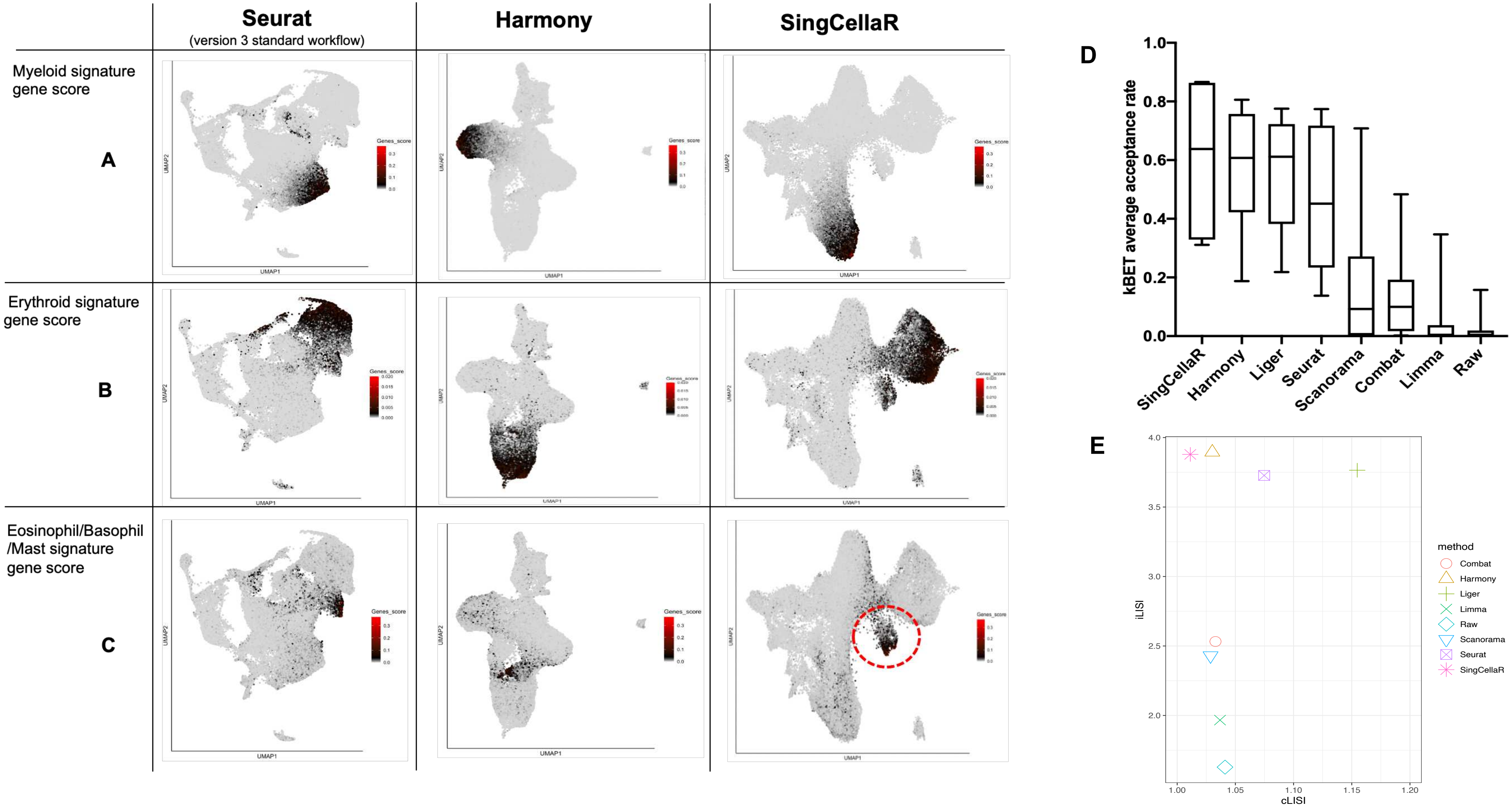


**Supplemental Figure 1. Optimal resolution of lineage progenitor clusters was obtained using a 'supervised Harmony' integration approach, implemented in SingCellaR (related to Figure 1).** UMAP plots of selected integration methods (Seurat, Harmony and SingCellaR) that performed well for this HSPC dataset. Lineage signature gene scores for (A) myeloid, (B), erythroid and (C) eosinophil/basophil/mast progenitors are superimposed on the UMAP plots, with a dashed circle highlighting the cluster of eosinophil/basophil/mast progenitor cells that is resolved only using SingCellaR. (D and E) Objective measures of integration for each method (D) Boxplot of kBET average acceptance rate score and (E) iLISI and cLISI scores. X-axis represents cLISI score. Y-axis represents iLISI score. A higher kBET (D) and iLISI score (E) indicates better data integration, and accurate integration should result in a higher iLISI score and cLISI score close to 1.

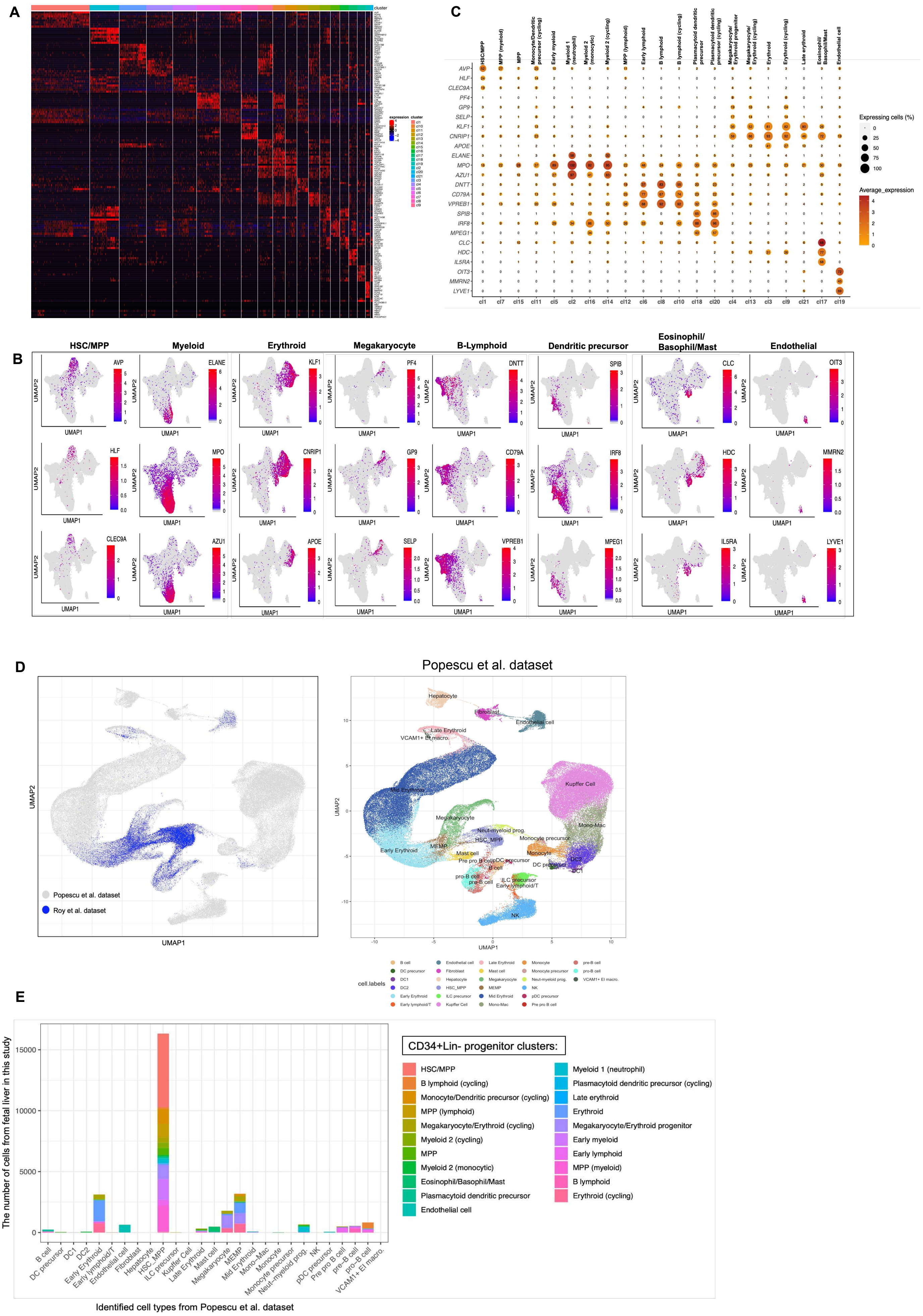


**Supplemental Figure 2. Identification of cell clusters using differentially expressed genes (related to Figure 1).** (A) Heatmap showing relative expression of the top 8 differentially expressed genes for each cluster. Cluster IDs are ranked from left (Cluster 1) to right (Cluster 21). (B) UMAP plots displaying the expression of canonical lineage marker genes. (C) Bubble plots showing the expression of lineage marker genes for each cluster. The size of the dot represents the percentage of cells expressing each gene (with the actual % shown inside the dot), and the colour represents the expression level. (D) Left panel – UMAP-plot displaying the mapping of fetal liver cells (from both 1st and 2nd trimester samples) from this study (Roy et al., blue dots) overlaid on top of the fetal liver reference dataset from Popescu et al. (grey dots) using the Symphony algorithm; Right panel – UMAP-plot displaying the annotated cell types in the Popescu et al. dataset. (E) Bar chart showing the mapping of fetal liver cells from this study on the annotated cell clusters from the Popescu et al. dataset.

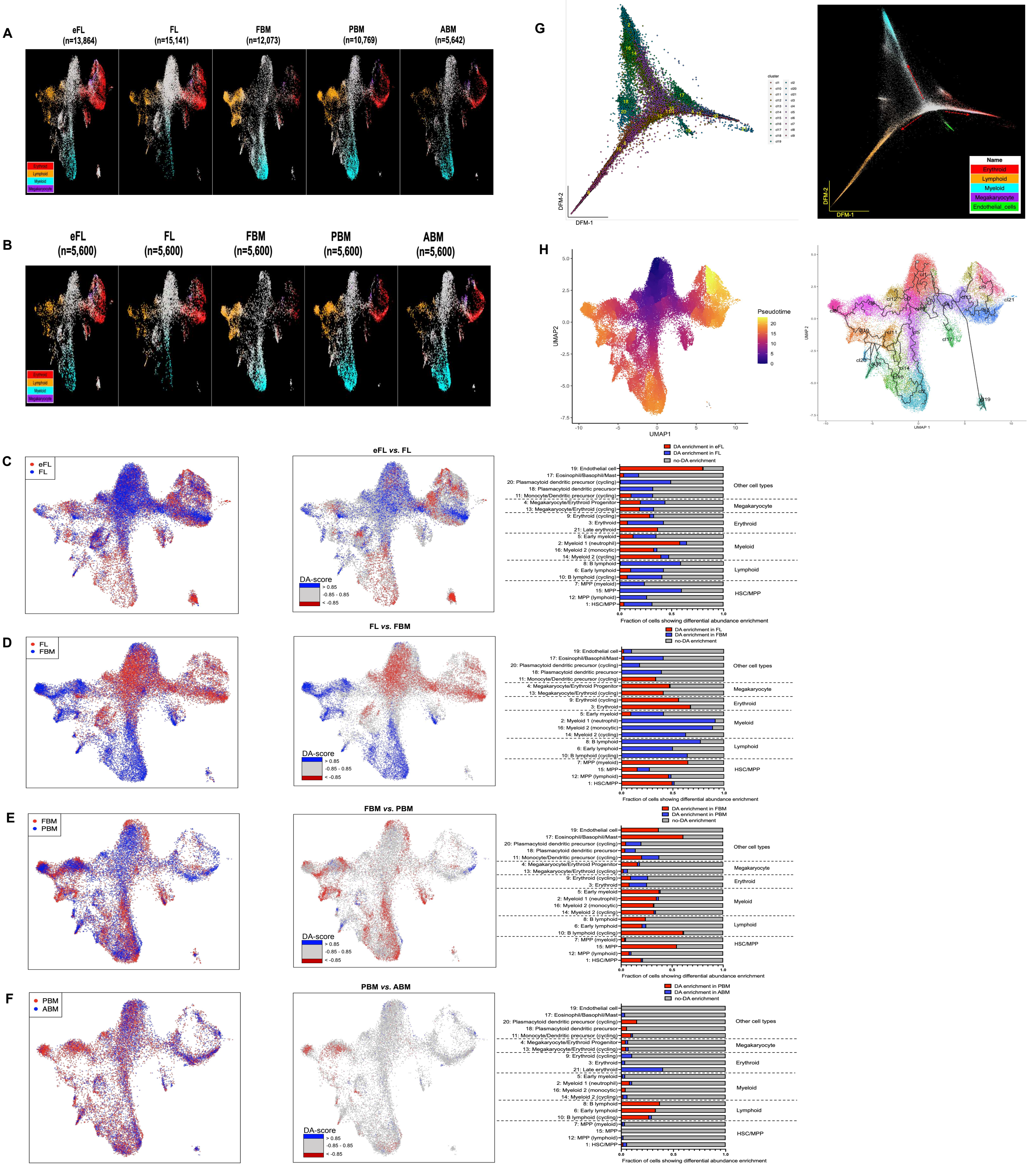


**Supplemental Figure 3. Dynamic changes in the cellular composition of HSPCs across tissues and differentiation trajectory analyses (related to Figures 2 and 3).** UMAP plots overlaid with lineage gene signature scores for HSC/MPP (grey); erythroid (red); lymphoid (yellow); myeloid (cyan) and megakaryocyte (purple) (A) showing all cells in each tissue (B) showing down-sampled cells (5600 cells per tissue). (C-F) Differential abundance (DA) analysis using DA-seq. Left panel, UMAP plots showing cells from pairwise comparisons of (C) eFL *vs.* FL, (D) FL *vs.* FBM, (E) FBM *vs.* PBM, and (F) PBM *vs.* ABM. Middle panel, UMAP plots showing the logistic classifier prediction for differentially abundant regions for each comparison. Cells in red are predicted to be more abundant in the first tissue with DA-score (using a cut-off score of < -0.85), whereas cells in blue indicate high abundance prediction in the second tissue (DA-score > 0.85). Cells in grey do not have a substantial DA score. Right panel, bar charts display the fraction of abundant cells per each cluster for each comparison. Red bar indicates the fraction of abundant cells per cluster in the first tissue. Blue bar indicates the fraction of cells in each cluster that have a high DA score, suggesting differential abundance in one tissue *vs.* the other. Grey bar indicates the fraction of cells in non-differentially abundant regions for each cluster. (G) HSPCs differentiation trajectories on a diffusion map. Left panel, diffusion map with cluster ID information. Right panel, diffusion map with the superimposition of 5 lineages progenitor gene sets. Red - erythroid; yellow - lymphoid; cyan - myeloid; purple - megakaryocyte; green - endothelial cells. The arrows indicate the main trajectories of cell differentiation from HSC/MPP towards lymphoid, erythroid/megakaryocyte, and myeloid differentiation. (H) Pseudotime and trajectory graph using Monocle3 analysis. Left panel, UMAP-plot superimposed with pseudotime scale. Dark-blue indicates the starting state and yellow or red indicates the terminal state of the cellular trajectories. Right panel, UMAP-plot with clusters and identified trajectory paths based on the monocle analysis.

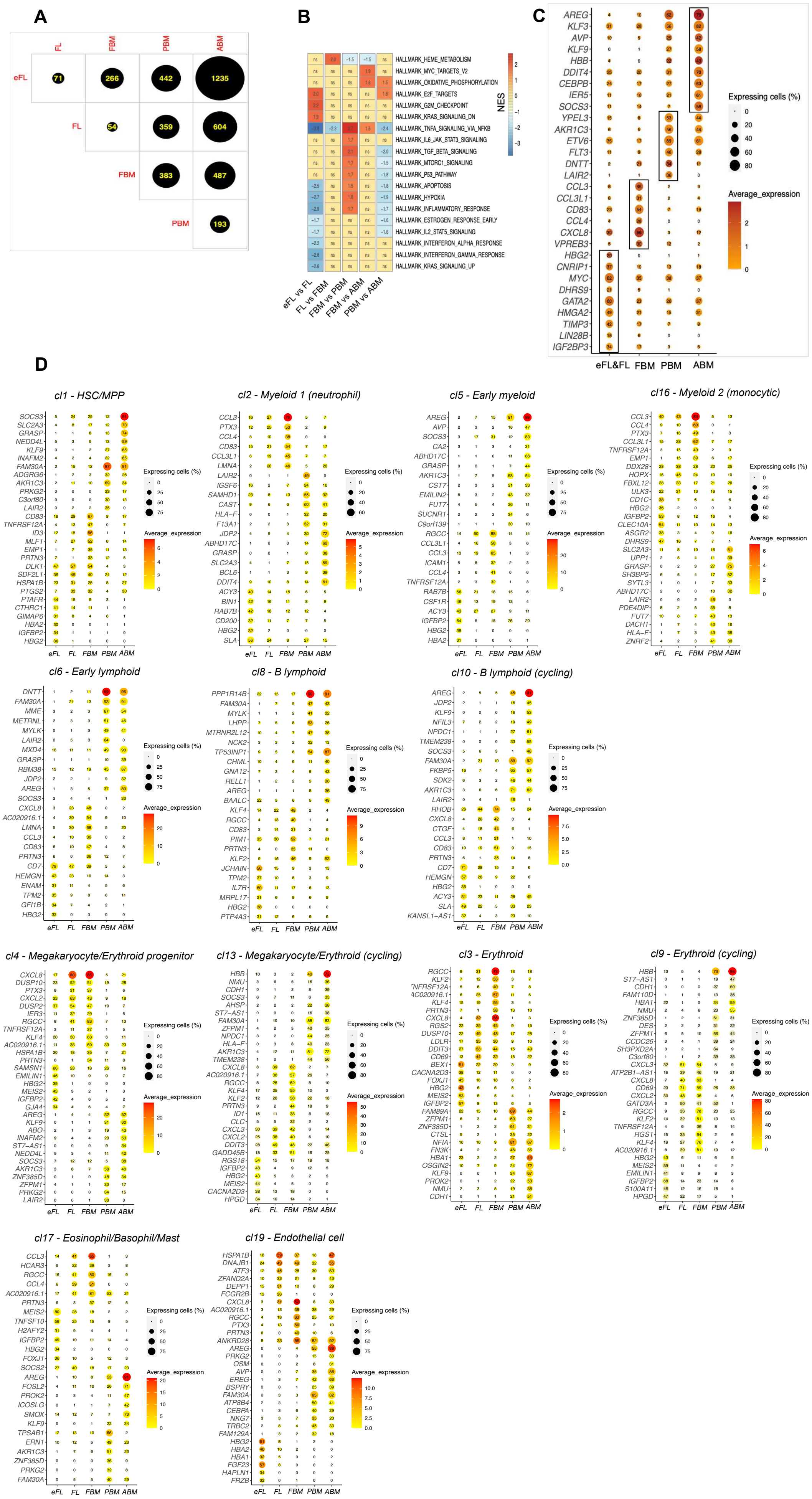


**Supplemental Figure 4. Dynamic changes in HSPC gene expression programs across human ontogeny (related to Figure 2 and STAR Methods – Quantification and Statistical Analysis).** (A) The number of differentially expressed genes between tissues in pairwise comparisons. (B) Heatmap of gene set enrichment scores for HALLMARK gene sets for the comparison of 5 selected pairs of tissues – eFL *vs.* FL, FL *vs.* FBM, FBM *vs.* PBM, FBM *vs.* ABM, and PBM *vs.* ABM. The gradient of colors represents the normalised enrichment score (NES) provided by GSEA. Red – gene sets enriched in the first tissue of the pair (positive values); blue – enrichment score for the second tissue of the pair (negative values). Yellow – no significant difference ('ns'). (C) Selected tissue-specific genes that are highly expressed in each tissue. (D) The top 6 significantly upregulated genes for selected lineage clusters for each tissue compared to all other tissues.

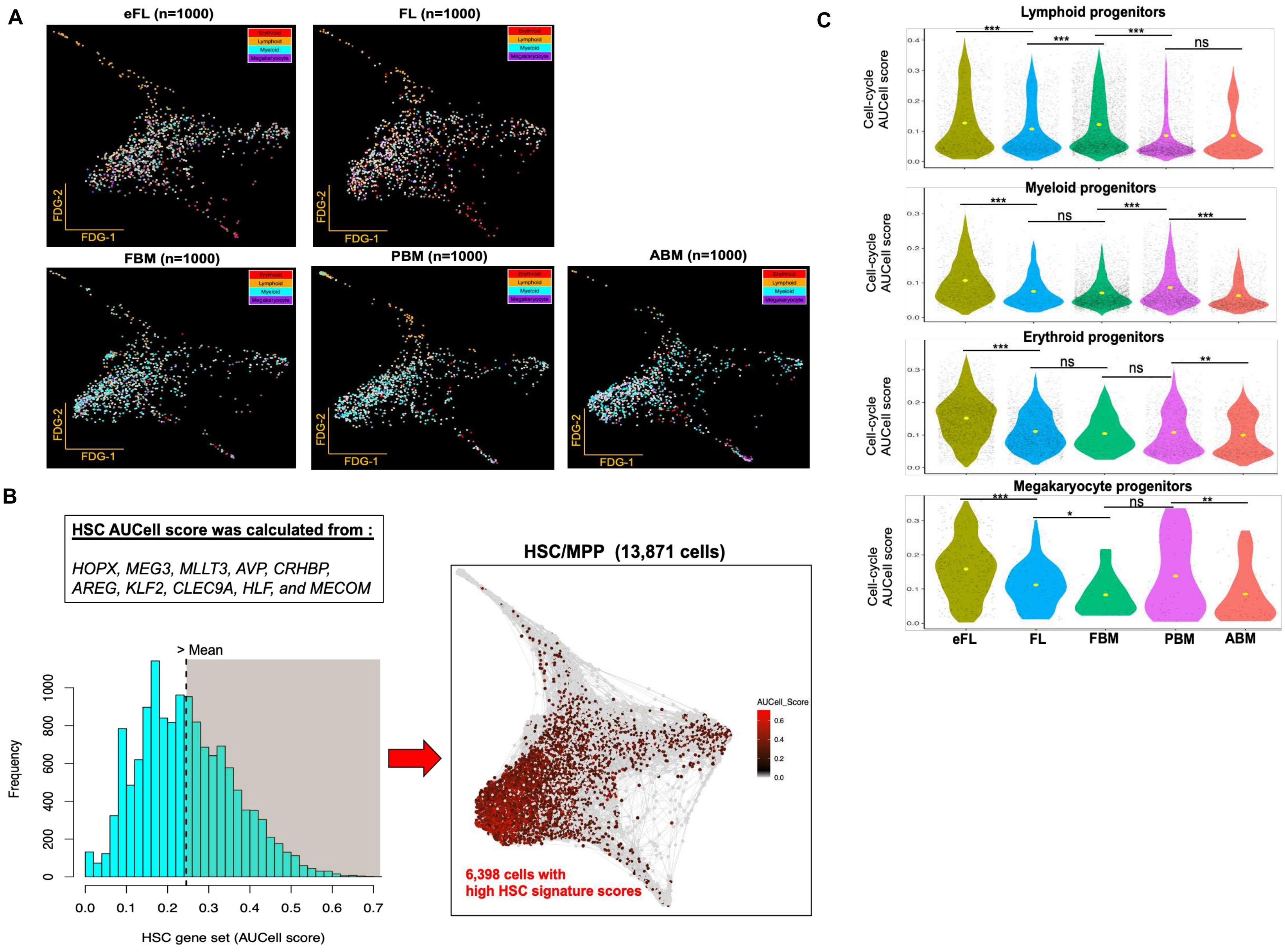


**Supplemental Figure 5. Selection of primitive HSCs and comparison of AUCell scores for selected gene sets across tissues (related to Figure 5).** (A) Force-directed graph trajectory analysis of selected HSC/MPPs across tissues following ‘down-sampling’ to 1,000 cells per tissue. Lineage signature gene expression is superimposed on top of the graph. Red - erythroid; yellow - lymphoid; cyan -myeloid; purple – megakaryocyte progenitors. (B) Left panel, the AUCell score distribution calculated from 10 curated HSC genes. 6,398 cells had higher HSC score than the average (>0.25) and were selected for downstream analyses. Right panel, force-directed graph represents selected HSC (black and red cells) in the trajectory of HSC/MPP compartment. (C) Violin plots comparing cell cycle AUCell scores in different lineages across tissues. Significance level shown as obtained using a Wilcoxon test (* = $P \leq 0.05$; ** = $P \leq 0.01$; *** = $P \leq 0.001$; and ‘ns’ = not significant).
