# Supplementary material for: Transitions in lineage specification and gene regulatory networks in hematopoietic stem/progenitor cells over human development
Source: Cell Rep. 2021 Sep 14;36(11):109698. doi: 10.1016/j.celrep.2021.109698 (PMC8456780; doi:10.1016/j.celrep.2021.109698)
Supplement: Document S2. Article plus supplemental information [file mmc9.pdf]

Cell Reports

Resource

Transitions in lineage specification and gene regulatory networks in hematopoietic stem/progenitor cells over human development

Graphical abstract

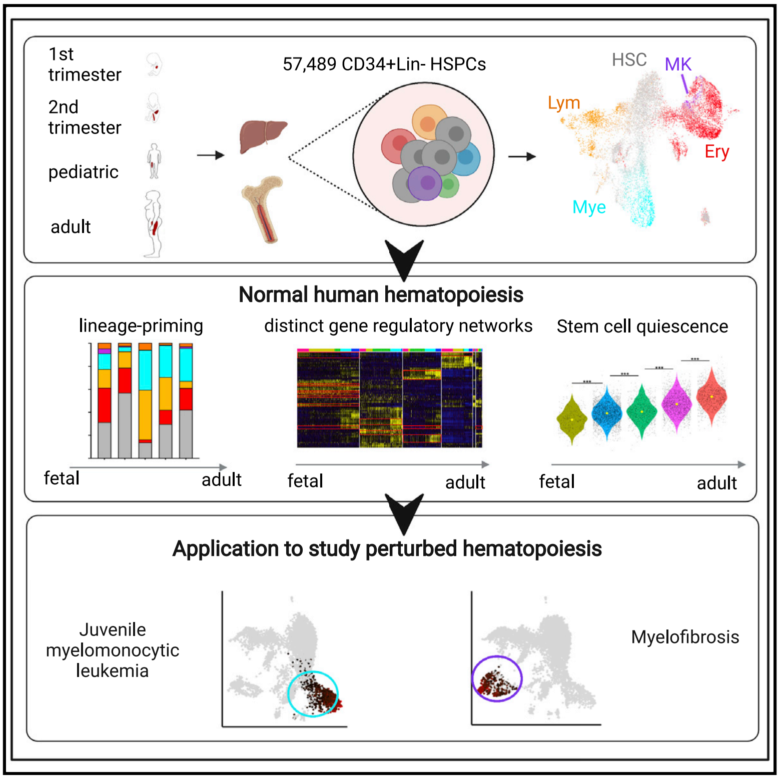

Highlights

- Sampling of >57,000 hematopoietic cells over 4 stages of human development
- Gene regulatory networks underpin changes in lineage specification through ontogeny
- Stem cells show progressive changes in cell cycling and inflammatory signaling
- Resource enables understanding of hematopoietic disorders presenting at specific age

Authors

Anindita Roy, Guanlin Wang, Deena Iskander, ..., Irene Roberts, Bethan Psaila, Supat Thongjuea

Correspondence

anindita.roy@paediatrics.ox.ac.uk (A.R.),
bethan.psaila@ndcls.ox.ac.uk (B.P.),
supat.thongjuea@imm.ox.ac.uk (S.T.)

In brief

Roy et al. use single-cell transcriptomics to compare lineage specification and gene regulatory networks of hematopoietic stem/progenitor cells (HSPCs) from multiple tissues over human ontogeny. Comparison to HSPCs from two blood cancers demonstrates the utility of the dataset for understanding disease processes and the emergence of age-specific blood malignancies.

Roy et al., 2021, Cell Reports *36*, 109698
September 14, 2021 © 2021 The Author(s).
https://doi.org/10.1016/j.celrep.2021.109698

CellPress

Cell Reports

CellPress OPEN ACCESS

Resource

Transitions in lineage specification and gene regulatory networks in hematopoietic stem/progenitor cells over human development

Anindita Roy,[1,2,3,7,*] Guanlin Wang,[2,4,7] Deena Iskander,[5] Sorcha O'Byrne,[1] Natalina Elliott,[1] Jennifer O'Sullivan,[2] Gemma Buck,[1,2] Elisabeth F. Heuston,[6] Wei Xiong Wen,[2,4] Alba Rodriguez Meira,[2,4] Peng Hua,[2] Anastasios Karadimitris,[5] Adam J. Mead,[2,3] David M. Bodine,[6] Irene Roberts,[1,2,3] Bethan Psaila,[2,3,7,*] and Supat Thongjuea[2,3,4,7,8,*]

[1]Department of Paediatrics, Children's Hospital, John Radcliffe Hospital, and MRC WIMM, University of Oxford, Oxford OX3 9DS, UK

[2]MRC Molecular Haematology Unit, MRC WIMM, University of Oxford, Oxford OX3 9DS, UK

[3]National Institute for Health Research (NIHR) Oxford Biomedical Research Centre, Oxford OX4 2PG, UK

[4]Centre for Computational Biology, Medical Research Council Weatherall Institute of Molecular Medicine (MRC WIMM), University of Oxford, Oxford OX3 9DS, UK

[5]Centre for Haematology, Department of Immunology and Inflammation, Imperial College London, London W12 0NN, UK

[6]Hematopoiesis Section, National Human Genome Research Institute, National Institutes of Health, Bethesda, MD 20892-4442, USA

[7]These authors contributed equally

[8]Lead contact

*Correspondence: anindita.roy@paediatrics.ox.ac.uk (A.R.), bethan.psaila@ndcls.ox.ac.uk (B.P.), supat.thongjuea@imm.ox.ac.uk (S.T.)

https://doi.org/10.1016/j.celrep.2021.109698

SUMMARY

Human hematopoiesis is a dynamic process that starts *in utero* 18–21 days post-conception. Understanding the site- and stage-specific variation in hematopoiesis is important if we are to understand the origin of hematological disorders, many of which occur at specific points in the human lifespan. To unravel how the hematopoietic stem/progenitor cell (HSPC) compartment changes during human ontogeny and the underlying gene regulatory mechanisms, we compare 57,489 HSPCs from 5 different tissues spanning 4 developmental stages through the human lifetime. Single-cell transcriptomic analysis identifies significant site- and developmental stage-specific transitions in cellular architecture and gene regulatory networks. Hematopoietic stem cells show progression from cycling to quiescence and increased inflammatory signaling during ontogeny. We demonstrate the utility of this dataset for understanding aberrant hematopoiesis through comparison to two cancers that present at distinct time points in postnatal life—juvenile myelomonocytic leukemia, a childhood cancer, and myelofibrosis, which classically presents in older adults.

INTRODUCTION

Definitive hematopoiesis begins in the human embryo at 4–5 weeks post-conception, when hematopoietic stem cells (HSCs) first arise in the aorta-gonad-mesonephros (AGM) region. Hematopoiesis then migrates to the fetal liver (FL) and subsequently to the bone marrow (BM), which becomes the dominant hematopoietic organ at birth and remains so throughout postnatal life (Ivanovs et al., 2017). Single-cell transcriptomics have been extensively applied to clarify the cellular architecture and molecular pathways in hematopoiesis, but the majority of studies have been conducted in adult tissues (Hay et al., 2018; Velten et al., 2017), mouse models (Dahlin et al., 2018; Tusi et al., 2018), or human cord blood (Notta et al., 2016; Zheng et al., 2018). A recent analysis of first and second trimester human FL, fetal kidney, and fetal skin indicated that the hematopoietic compartment in FL changes from being predominantly erythroid in early gestation to lympho-myeloid in later development, and that hematopoietic stem/progenitor cells (HSPCs) become less proliferative during fetal maturation (Popescu et al., 2019). The transition of HSPCs from a proliferative to quiescent state has also been associated with the migration of hematopoiesis from FL to fetal BM (FBM), along with a concomitant decrease in the frequency of non-committed HSPCs, suggesting a role for the niche in regulating hematopoietic cell state (Ranzoni et al., 2021).

However, the transcriptome profiles of the lineage-negative (Lin^-), $CD34^+$ HSPC compartment in hematopoietic tissues during development and postnatal aging have never been directly compared using the same platform. These studies are crucial if we are to understand the variation in hematopoiesis during normal human ontogeny. For example, whether certain patterns of fetal, pediatric, or adult-specific gene expression signatures exist that are permissive for the emergence of age-dependent hematopathologies has not previously been described.

We therefore generated a comprehensive dataset encompassing 57,489 HSPCs sampled from healthy human

This is an open access article under the CC BY license (http://creativecommons.org/licenses/by/4.0/).

hematopoietic tissues, including first trimester early FL (eFL), paired second trimester FBM and FL (isolated from the same fetuses), pediatric BM (PBM), and adult BM (ABM). This study directly compares human HSPC from all stages of ontogeny (early fetal life to adulthood) at the single-cell level. Precise delineation of the consistencies and differences in the cellular composition and molecular pathways in the HSPC compartments across human development demonstrated pronounced site- and developmental stage-specific transitions in cellular architecture and transcriptional profiles between hematopoietic tissues. While megakaryo-erythropoiesis predominated in eFL, lympho-myeloid progenitors showed dramatic expansion following the onset of hematopoiesis in the BM of the developing fetus. The proportion of lymphoid progenitors in the BM hematopoietic compartment then progressively decreased during postnatal life. Analysis of the most naive HSPCs suggested that molecular programs that underpinned these "shifts" in lineage composition originated very early within the primitive HSC compartment. In addition, HSCs but not lineage-committed progenitors showed progression from cycling to quiescence and increased inflammatory signaling during development from fetal through to adult life. Finally, comparison of HSPCs sampled over normal human ontogeny to HSPCs from two hematological cancers that occur at extremes of life, juvenile myelomonocytic leukemia (JMML) affecting young children and myelofibrosis (MF) affecting older adults, suggested a fetal origin for JMML and exacerbation of the adult BM-associated inflammatory signaling programs in MF HSPCs. These observations highlight the value of this comprehensive single-cell transcriptomic resource for understanding the changes in normal hematopoiesis through human ontogeny, as well as unraveling the cellular and molecular disruptions that occur in hematopoietic disorders.

RESULTS

Analysis of 57,489 HSPCs revealed 21 distinct cell clusters across human ontogeny

Single-cell RNA sequencing (scRNA-seq) was performed on Lin^{-}CD34+ cells from: eFL (15,036 cells); matched FL (17,351 cells) and FBM (14,935 cells) isolated from the same fetuses; PBM (13,311 cells); and ABM (6,300 cells) using the 10x Genomics platform (Figure 1A). A total of 66,933 HSPC were captured, and following quality control, 57,489 single-cell transcriptomes were included in downstream analyses (Table S1). Datasets were integrated using an adaptation of the Harmony algorithm (Korsunsky et al., 2019) that automatically finds the similarity of identified clusters across all samples by performing sequential gene set enrichment analysis (GSEA) with identified marker genes from differential gene expression analysis (see STAR Methods), implemented in the SingCellaR package. This led to optimal data integration with improved resolution of erythroid from eosinophil/basophil/mast progenitors than when using the standard Harmony or Seurat methods as indicated by visual inspection (Figures S1A–S1C) and the objective measures kBET, iLISI, and cLISI (Figures S1D and S1E) (Büttner et al., 2019; Korsunsky et al., 2019).

To identify the cellular composition of the 57,489 HSPCs captured from different stages and tissues over ontogeny, graph-based Louvain clustering was performed. This identified 21 clusters with distinct expression patterns of canonical stem/progenitor and hematopoietic lineage marker genes (Figures 1B, S2A−S2C; and Table S2) (Drissen et al., 2019; Hay et al., 2018; Pellin et al., 2019; Popescu et al., 2019; Velten et al., 2017). While some clusters were easy to identify by their clear expression of canonical markers, other clusters were less readily classifiable due to the low-level expression of lineage-affiliated genes and/or expression of multiple lineage markers. To facilitate the annotation of these clusters, we collated lineage signature genes from 75 published human hematopoiesis gene sets (Table S3) and performed GSEA on the expressed genes for each cluster using these gene sets to guide assignment of cell types (Figure 1C).

Cluster 1 had a robust expression of hematopoietic stem cell/multipotent progenitor (HSC/MPP)-affiliated genes, while clusters 7 and 12 showed HSC/MPP genes together with evidence of early myeloid or lymphoid priming, respectively (Figures 1B and 1C, and Figure S2C). Cell clusters representing the major hematopoietic lineage progenitor subsets (erythroid, myeloid, lymphoid, megakaryocytic, dendritic, eosinophil/basophils/mast cells) were identified, as well as clusters with the expression of genes representing >1 lineage likely to represent non-lineage-primed HSC/MPP or oligopotent progenitors (e.g., erythro-megakaryocytic [cluster 4] and eosinophil/basophil/mast cell precursors [cluster 17]) (Figure 1C). A population of non-hematopoietic cells (cluster 19) expressing endothelial genes was detected, which derived predominantly but not exclusively from eFL. Some lineage progenitor clusters had differential enrichment of G2M checkpoint and S phase gene signatures (Table S4), allowing us to classify certain progenitor populations by their distinct proliferation state (e.g., cluster 9, erythroid [cycling]; cluster 10, B lymphoid [cycling]; Figure 1B). To further investigate the resolution of this dataset, we compared our fetal liver data with the published scRNA-seq data from FL (Popescu et al., 2019). The FL cells from our study mostly mapped to CD34^{+} progenitor/precursor cell clusters of the reference dataset (HSC/MPP, PreProB, ProB, MEMP, early erythroid, DC precursors, and neutrophil-myeloid progenitors) (Figures S2D and S2E). As expected, given the sorting strategy that we used, our dataset achieved a higher resolution of cell populations within the HSC/MPP and progenitor populations (Figures S2D and S2E).

Site- and developmental stage-specific differences in the cellular composition of the HSPC compartment

To accurately determine differences in the composition of Lin^{-}CD34^{+} progenitors between hematopoietic sites and ontological stages, we identified cells showing an enriched expression of curated gene signatures corresponding to HSC/MPP and to the main lineage progenitor subtypes (myeloid, mega-erythroid-mast cell, and B-lymphoid progenitors; Figure 2A; Table S4). Using an AUCell (Aibar et al., 2017) score of >0.15, a clear distinction between cellular subsets was seen, with 24% of cells (13,871 cells) classified as HSC/MPP, 15% as myeloid (8,330 cells), 16% as lymphoid (9,473 cells), 2% as eosinophil/basophil/mast cell (1,361 cells), 12% as erythroid (6,778 cells), and 1% as megakaryocytic (572 cells) progenitors (Figure 2A).

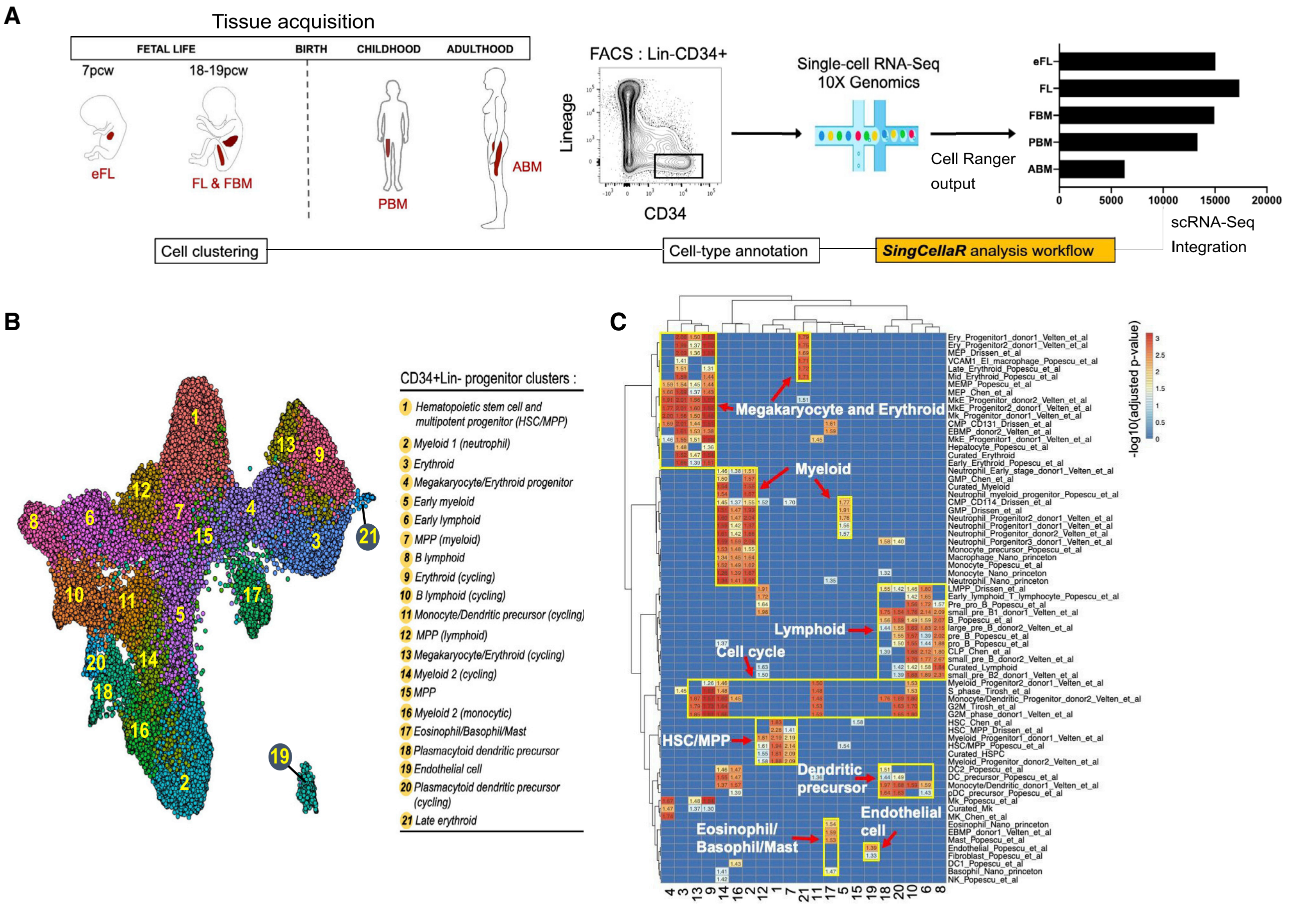

Figure 1. Single-cell RNA-sequencing (scRNA-seq) of 57,489 hematopoietic stem/progenitor cells (HSPCs) from 5 hematopoietic tissues across human ontogeny reveals 21 distinct cellular subsets

(A) Experimental design. Lineage-negative (Lin$^-$) CD34$^+$ HSPCs were fluorescence-activated cell sorting (FACS) sorted for scRNA-seq from first trimester (early) fetal liver (eFL), matched second trimester FL and fetal bone marrow (FBM), pediatric bone marrow (PBM), and adult bone marrow (ABM). A bar chart shows the number of cells per tissue.

(B) Louvain community-detection clustering based on the weighted graph network and uniform manifold approximation and projection (UMAP) of 57,489 cells identified 21 distinct hematopoietic progenitor populations.

(C) Heatmap generated using the SingCellaR cell-type annotation system showing positive gene set enrichment scores for each cluster facilitates cluster identification. The x axis represents clusters as numbered in Figure 1B. The y axis represents a curated list of hematopoietic lineage-specific gene sets (see Table S3).

First and second trimester FL contributed the majority (69.4%) of the erythroid progenitors captured (Figure 2B), whereas lymphoid and myeloid progenitors predominantly derived from BM samples (70.4% and 81.2% respectively; Figure 2B). Marked differences were observed in the origin of the megakaryocyte progenitor cells captured, with a majority (63.5%) captured from eFL (Figure 2B), while only 5%–12% derived from any other individual tissue. Differences in lineage specification were also demonstrable when the proportions of progenitor cell types were quantified within individual tissue (Figures 2C and S3A). These differences in lineage specification for the main lineage subtypes described above were maintained after adjusting for the variable cell numbers obtained from each tissue type (Figure S3B). Of note, marked differences were seen in the cellular composition of matched FL and FBM HSPCs when analyzing cells representing HSC/MPP and the main lineage subtypes. There were 4.4-fold more HSC/MPP and 8.7-fold more erythroid progenitors in FL than FBM. The opposite trend was true for myeloid and lymphoid progenitors, which were 7.9-fold and 2.8-fold more frequent in FBM than FL (Figure 2C). The observations in regard to the shifts in composition of the HSPC compartment over ontogeny were confirmed using DA-seq, a differential abundance analysis method that enables comparative analyses of different samples independently of clustering analysis (Zhao et al., 2020). Comparison of FL to FBM showed a strong enrichment of lympho-myeloid progenitors in FBM and HSC/MPP, MPP, and mega-erythroid progenitors in FL. Comparison of FBM to PBM showed enrichment of lympho-myeloid progenitors in FBM, and PBM to ABM highlighted the enrichment of lymphoid progenitors in PBM (Figures S3C–S3F). These results confirm our observations from the clustering analyses.

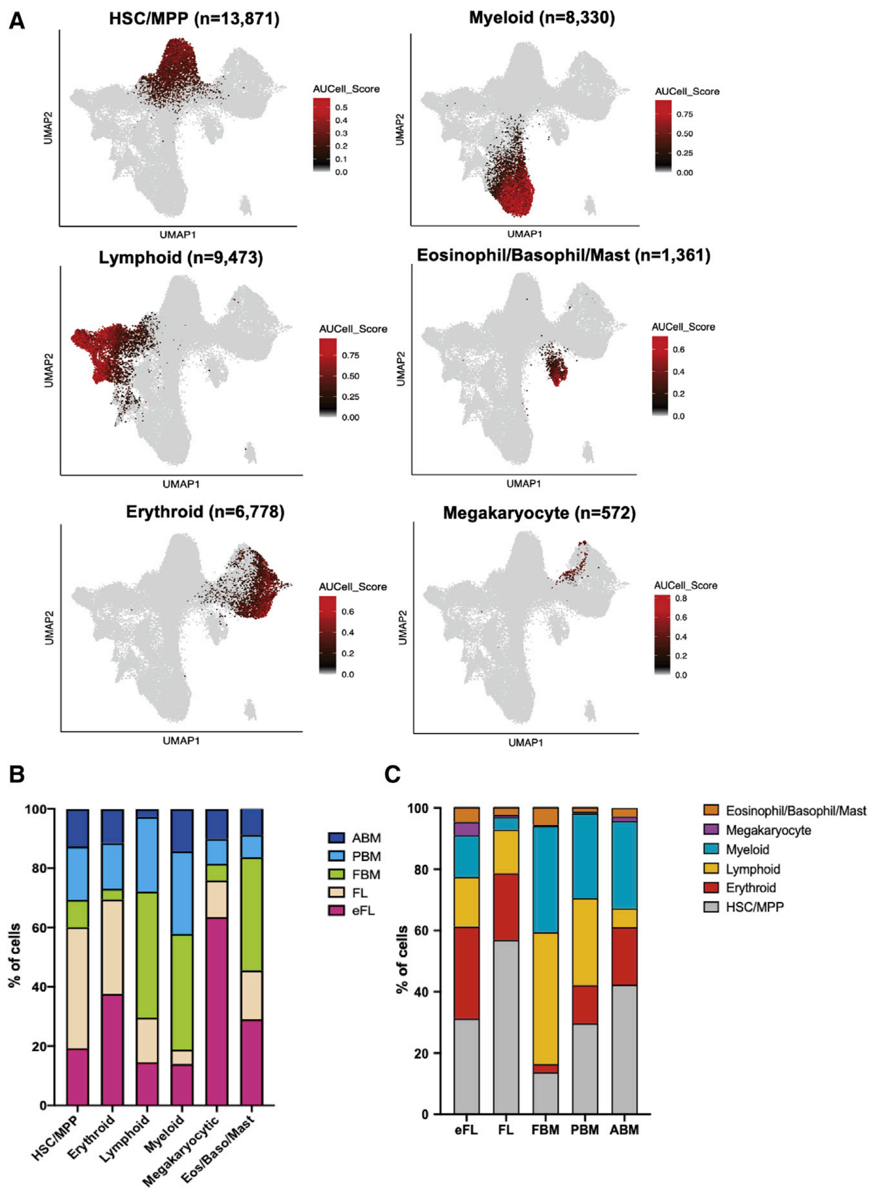

Figure 2. Site and developmental stage-specific changes in composition of the HSPC compartment

(A) Cells were classified using an AUCell score of >0.15 for canonical marker genes for HSC/MPP, myeloid, lymphoid, eosinophil/basophil/mast cell, erythroid, and megakaryocyte progenitors.

(B) The proportion of HSC/MPP and lineage progenitor subsets that derived from each tissue type.

(C) The proportion of HSPCs from each tissue classified as HSC/MPP or lineage progenitor subtypes.

Four main differentiation trajectories are present among HSPCs across human ontogeny

To explore the developmental relationships between cell clusters, cells were ordered based on their gene expression using a force-directed graph (FDG) network (Figures 3A–3C). Lineage signature gene scores were defined as before (Psaila et al., 2020) and superimposed on the FDG. This demonstrated four major paths emerging from the HSC/MPP cluster (cluster 1, Figure 3A), representing erythroid, megakaryocytic, lymphoid, and myeloid differentiation trajectories along pseudotime (Figures 3B and 3C). As expected, clusters with multipotent potential (clusters 1, 5, 7, 11, 12, and 15) were located at the apex or central positions in the trajectories (Figures 3A and 3B, gray cells). The existence of the main trajectories was confirmed using diffusion mapping as an alternative trajectory analysis (Figure S3G).

Dendritic cell precursors (clusters 18 and 20) were closely affiliated with the lymphoid trajectory, while the trajectory of eosinophil/mast cell/basophil progenitors (cluster 17) was associated with that of erythroid progenitors (Figures 3A and 3B).

Monocle3 (Trapnell et al., 2014) analysis was performed to calculate pseudotime and identify differentiation paths for the four main trajectories (erythroid, megakaryocytic, lymphoid, and myeloid) (Figure S3H). To compare lineage differentiation trajectories across tissue types, the datasets were downsampled to control for differences in the numbers of cells captured from each tissue, and cells were then ordered in pseudotime (Figures 3D–3K). The expression of erythroid and megakaryocyte-associated transcriptional programs occurred earlier in pseudotime for eFL than for other tissues (Figures 3D–3G). The expression of lymphoid-associated genes was markedly different between tissues across ontogeny, with the onset of expression observed earliest in BM hematopoiesis in the second trimester fetus, and then progressively later in PBM and ABM cells (Figures 3H and 3I). This included both early lymphoid genes and later B cell-specific genes, suggesting an accelerated B cell specification program in fetal and pediatric BM. Myeloid-associated gene expression occurred earlier in pseudotime for all BM tissues compared to eFL/FL and was particularly pronounced in FBM (Figures 3J and 3K). The differences between matched FL and FBM from the same donors highlight differences in lineage specification between hematopoietic sites at the same developmental stage (Figures 3D, 3F, 3H, and 3J).

Developmental stage drives global transcriptional differences between HSPCs, with enrichment of cell-cycle genes in eFL and inflammatory pathways in ABM

HSPCs from different developmental stages and hematopoietic sites showed distinct molecular profiles. A total of 4,094 genes

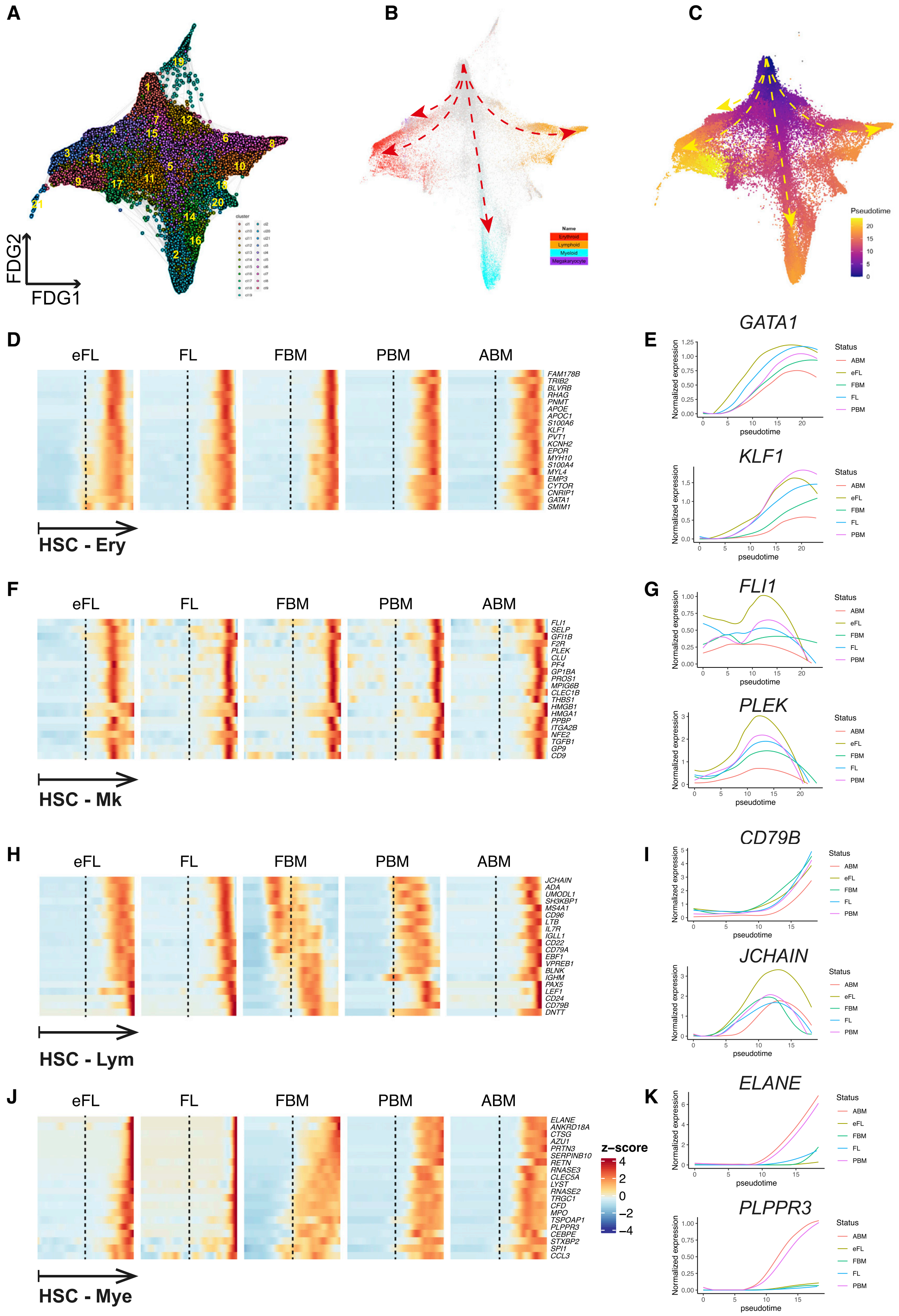

(legend on next page)

was identified as differentially expressed genes (DEGs) upon pairwise comparison of the 5 tissue types, with the largest number of DEGs found between eFL and ABM (1,235 DEGs) and the smallest difference between FL and FBM samples from the same developmental stage (54 DEGs), suggesting that developmental stage is a more significant driver of global transcriptional differences than is the site of hematopoiesis (Figure S4A). Hallmark GSEA on DEGs between pairwise comparisons of selected tissues demonstrated the enrichment of cell-cycle-related pathways (e.g., E2F target and G2M checkpoint) in eFL versus FL, heme metabolism in FL versus FBM, and multiple inflammatory response-related pathways in adult versus pediatric BM (Figure S4B).

We also identified subsets of specific genes that were differentially expressed in each tissue when compared to all of the others (Figure S4C;Table S5). Unsurprisingly, genes involved in controlling the cell cycle (*MYC)* and erythroid lineage-associated genes (*CNRIP1*, *TIMP3*, and *GATA2*) together with fetal-specific genes (*LIN28B* and *IGF2BP3*) were highly expressed in eFL and FL (Copley et al., 2013; McWilliams et al., 2013; Yuan et al., 2012). *DHRS9*, a robust marker for regulatory or suppressive macrophages (Riquelme et al., 2017), was also specifically expressed in FL. FBM had a strong expression of chemokine ligands (*CCL3*, *CCL3L1*, *CCL4*, and *CXCL8*) as well as B lymphoid genes (*VPREB3* and *CD83*) (O'Byrne et al., 2019), reflecting the strong myeloid/lymphoid skew in FBM. The postnatal samples PBM and ABM had relatively similar transcriptional profiles, with only 193 genes being DEGs (Figure S4A). However, the expression of *LAIR2*, a gene encoding an immunoglobulin superfamily receptor, was very specific for PBM, perhaps associated with its role in the establishment of the immune system during childhood (Figure S4C). Other genes expressed more highly in PBM versus ABM included genes associated with early B-lymphoid development (*DNTT* and *FLT3*), the transcription factor and proto-oncogene (*ETV6*) that is frequently mutated in hematological malignancies, and genes involved in cell growth, proliferation, and apoptosis (*YPEL3* and *AKR1C3*; Figure S4C). ABM strongly expressed kruppel-like factors (*KLF3* and *KLF9)*, markers of DNA stress (*DDIT4*), a regulator of inflammation (*SOCS3*), the protein phosphatase *IER5* that regulates cell growth and stress resistance (Kawabata et al., 2015), and myeloid-associated genes (*AREG* and *CEBPB*) compared to other tissues (Figure S4C). As expected, the switch from fetal to adult hemoglobin was also evident with the fetal gamma globin gene *HBG2* strongly expressed in FL in contrast to PBM and ABM, where the beta globin gene (*HBB*) was the most prominently expressed beta globin cluster gene. A complete list of significantly DEGs compared across tissues is provided in Table S5, and tissue-specific genes per each cluster are included in Table S6. The top 6 significantly upregulated genes in each tissue for each cluster are shown for 13 selected clusters (Figure S4D).

Distinct gene regulatory networks (GRNs) in HSC/MPP underlie differences in lineage specification between hematopoietic tissues

To examine the molecular regulators that underpin the differences in lineage priming and cellular heterogeneity between hematopoietic tissues, we performed single-cell regulatory network inference and clustering (SCENIC) to evaluate the activity of GRNs (Aibar et al., 2017). This method enables identification of regulons, or genes that are co-expressed with transcription factors, with known direct binding targets based on *cis*-regulatory motif analysis. The activity score of each regulon was quantified in each cell using AUCell (Figure 4A). This enabled the identification of regulons that were specifically enriched in HSC/MPP (ZNF467, HOXA9, and HOXB5), lymphoid progenitors (PAX5, TCF3, and TBX21), myeloid progenitors (SPI1, CEBPD, and RUNX1), erythroid progenitors specifically (HES6), megakaryocytic-erythroid progenitors (STAT5A and GATA1), eo/baso/mast cell progenitors (FEV and FOXD4L1), and megakaryocytic progenitors (MEF2C) (Figure 4A).

The regulon activity was compared across tissue types for each HSPC subset (Figure 4B). While the main differences in regulons were driven by the lineage specification of HSPC, there were tissue-specific differences between tissues within HSC/MPP and lineage-primed progenitor compartments. HSC/MPP from eFL had stronger enrichment of MYC, HDAC2, HMGA1, FLI1, and MEF2C, and the expression of these regulons decreased substantially during development, suggesting a key role for these GRNs in promoting erythroid and megakaryocytic differentiation in eFL versus hematopoietic tissues later in normal human development (Figure 4B). Fetal HSC/MPP also showed enrichment for HOXA9 and HOXB5 regulons compared to postnatal tissues. There was an enrichment of lymphoid-associated GRNs in HSC/MPP from PBM, and this was accompanied by the strong enrichment of ZMIZ1, IKZF1, BCL11A, and ETS1 regulons in PBM lymphoid progenitors. TBX21, WT1, and SOX4 regulons were enriched in eFL lymphoid progenitors; MEF2D, SPIB, IRF4, IRF7, IRF8, RUNX1, and CEBPA/D/E in postnatal myeloid progenitors; and MYC, HDAC2, and HMGA1 in fetal versus postnatal erythroid progenitors. RAD21 and CTCF targets were specifically enriched in postnatal versus prenatal HSC/MPP, erythroid, and eo/baso/mast cell progenitors (Figure 4B).

Differences in the transcriptome of HSC/MPP through ontogeny

We next sought to investigate differences in the gene expression patterns of more naive HSPCs (HSC/MPP) through ontogeny, to

Figure 3. Four main differentiation trajectories were identified, and onset of lineage-affiliated transcriptional programs varied along "pseudotime" between tissues

(A–C) Force-directed graph (FDG) showing (A) 21 Louvain clusters, (B) superimposition of gene scores for 4 lineage gene sets, (C) pseudotime score from Monocle3 analysis. Dashed arrows represent the 4 main trajectories of differentiation from HSC/MPP toward lymphoid, erythroid, megakaryocytic, and myeloid progenitors.

(D, F, H, and J) Expression of canonical lineage-affiliated genes along pseudotime from HSC to erythroid, megakaryocytic, lymphoid, and myeloid trajectories for each tissue.

(E, G, I, and K) Expression of selected erythroid, megakaryocytic, lymphoid, and myeloid lineage-affiliated genes along pseudotime for each tissue.

CellPress
OPEN ACCESS

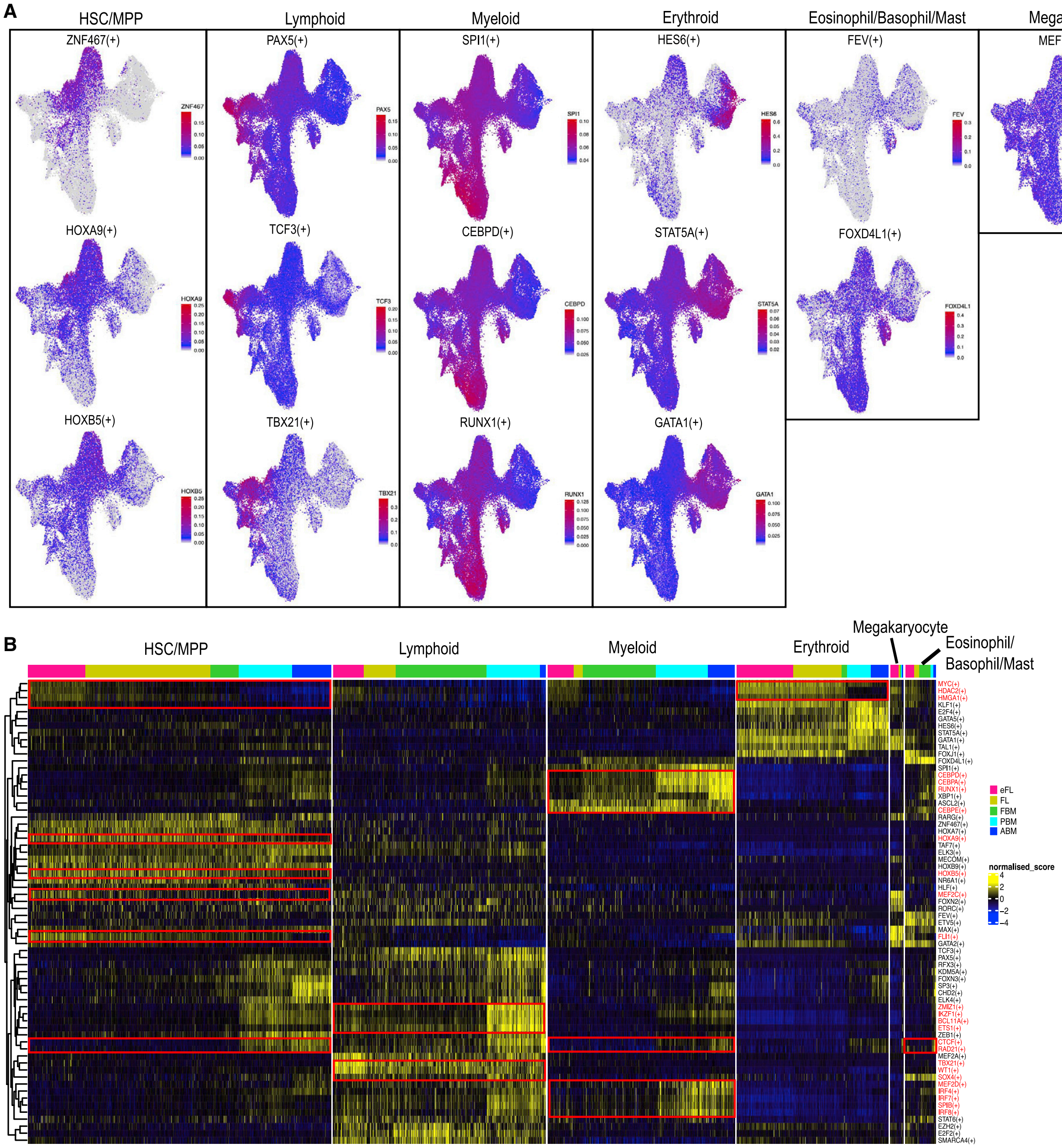

Figure 4. Distinct activity of gene regulatory networks (GRN) underlies differences in lineage specification across ontogeny

(A) UMAP plots superimposed with enrichment scores from SCENIC analysis for regulons specifically enriched in each lineage. HSC/MPP (ZNF467, HOXA9, HOXB5); lymphoid (PAX5, TCF3, TBX21); myeloid (SPI1, CEBPD, RUNX1); erythroid specific (HES6); mega-erythroid (STAT5, GATA1); eo/baso/mast cell (FEV, FOXD4L); and megakaryocyte specific (MEF2C).

(B) Heatmap showing selected regulons that showed differential activity between lineages across tissues. The red boxes and names indicate regulons highlighted in the main article.

identify possible cell-intrinsic factors underlying the changing differentiation bias during development. Cells from all of the tissues that were classified as HSC/MPP using an AUCell score >0.15 were selected for further analysis (Figures 5A and 5B). This demonstrated four distinct cell clusters: two minor subfractions that showed low-level enrichment of mega-erythroid and lympho-myeloid lineage gene signature sets, suggesting early lineage priming of some HSC/MPP (Figure 5C), and the non-lineage primed HSC/MPP were separated into two distinct subsets reflecting proliferative and quiescent cell states (Figures 5D and 5E). After downsampling the data to analyze equal numbers of HSC/MPP from each tissue type, BM HSC/MPP from all of the developmental stages (FBM, PBM, and ABM) showed myeloid priming compared to FL samples, and PBM HSC/MPP were

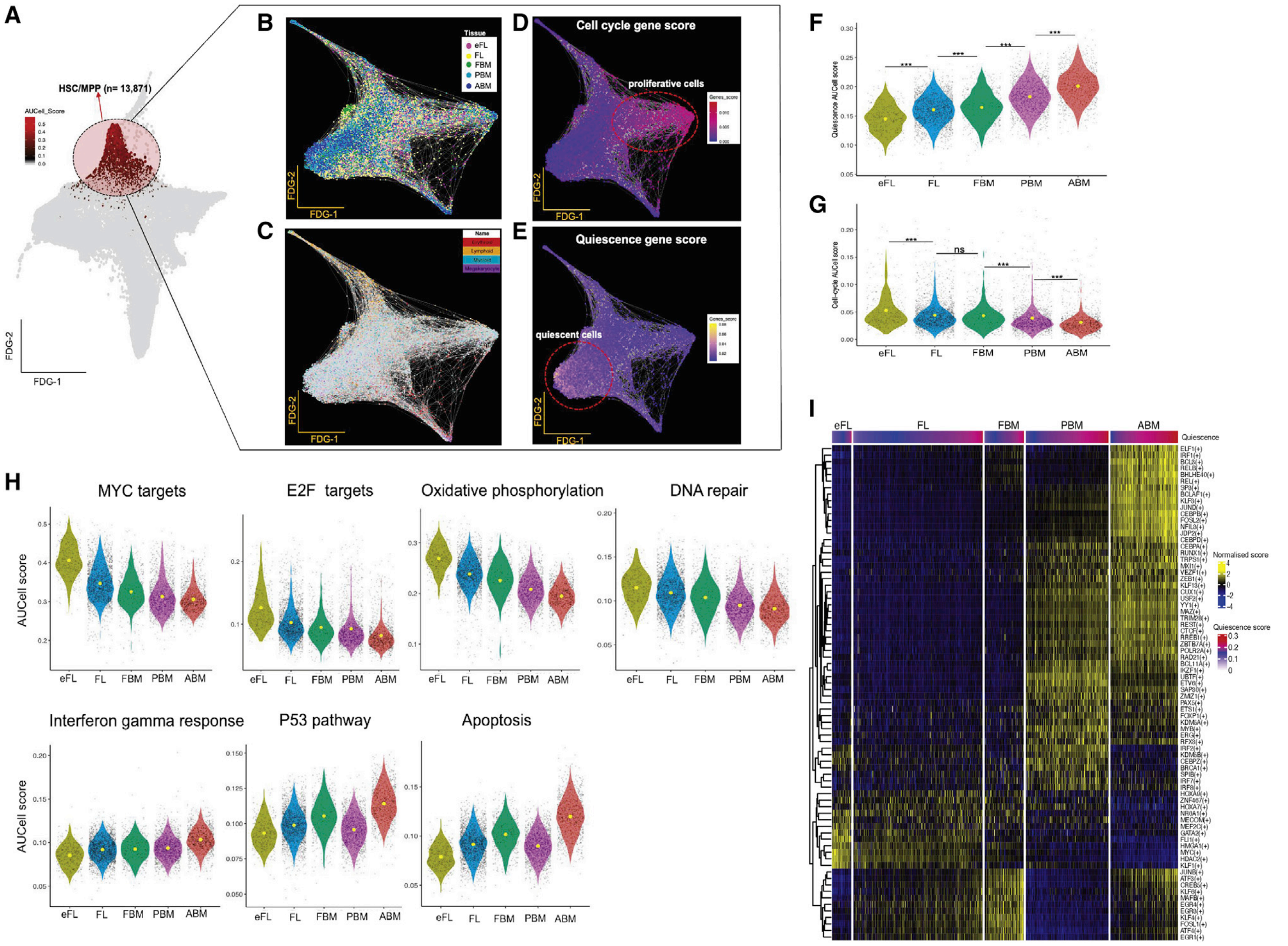

Figure 5. HSC/MPPs show early lineage priming and progression from cycling to quiescence over ontogeny, with differential GRN activity between tissues

(A) A total of 13,871 cells were identified as HSC/MPP using an AUCell score >0.15 for the HSC gene set and visualized in a FDG with superimposition of (B) tissue of origin.

(C) Lineage gene set.

(D) Cell-cycle gene expression scores.

(E) Quiescent gene expression scores.

(F and G) AUCell scores for quiescence and cell-cycle signature gene sets in a total of 6,398 HSC identified from different tissues. Significance level shown as obtained using a Wilcoxon test (*p $\leq$ 0.05; **p $\leq$ 0.01; ***p $\leq$ 0.001; and ns, not significant).

(H) Violin plots comparing AUCell scores from each selected HALLMARK gene set across tissues.

(I) Differentially active regulons in HSC across tissues. Cells for each tissue were ranked by the quiescence score.

more lymphoid primed than ABM, similar to our observations in total Lin^{-}CD34^{+} HSPCs (Figure S5A).

HSCs show progression from proliferation to quiescence during ontogeny with enrichment of inflammatory signatures in ABM

To investigate the transcriptional profile of the most primitive HSC, cells with the very highest HSC AUCell score (greater than mean score) within the HSC/MPP compartment were taken forward for further characterization (6,398 cells; Figure S5B). Quiescence and proliferation gene signatures (Table S4) showed opposite changes through ontogeny, with ABM HSC showing the highest quiescence score and eFL HSC being the most proliferative (Figures 5F and 5G). Notably, this temporal pattern in cell cycling was only observed in HSCs, and cell-cycle scores in other lineage-affiliated progenitor clusters did not show the same progression across ontogeny (Figure S5C). The decrease in cell-cycle score through ontogeny correlated with the decreasing expression of MYC and E2F targets, oxidative phosphorylation, and DNA repair pathways. In contrast, increasing HSC quiescence correlated with inflammatory response, activation of P53 response, and cell death-associated pathways (Figure 5H).

We next sought to determine whether cell-intrinsic drivers of lineage priming and cell cycling were detectable in the most primitive HSCs. We therefore examined the regulons that were

actively enriched in the 6,398 HSCs with the highest HSC AUCell score at different stages of ontogeny. eFL and FL HSC showed evidence of erythroid- and megakaryocyte-specific regulons such as KLF1, GATA2, FLI1, and MEF2C (Figure 5I). The FBM regulon was enriched for calcineurin-regulated nuclear factor of activated T cells (NFAT)-dependent transcription networks (FOSL1, JUNB, EGR3, EGR4, and MAFB) that are usually seen in lymphocytes, which may represent early lymphoid priming. PBM HSC showed a predominance of B lymphoid-specific regulons, such as BCL11A, IKZF1, PAX5, and IRF8 compared to other tissues. The regulons specifically expressed in ABM HSCs were predominantly mediating inflammatory programs (e.g., interferon pathway [ELF1 and IRF1], NFKB, and acute phase response [CEBPB], as well as genes previously reported as deregulated in aged HSCs [FOSL2 and JUND]; Figure 5I) (Lavrovsky et al., 2000; Schäfer et al., 2018).

Leveraging single-cell transcriptomics to understand abnormal hematopoiesis

Finally, to demonstrate the utility of the dataset for understanding transcriptional perturbations in hematological diseases, we compared the normal ontogeny dataset to previously published single-cell datasets from two disease states. We chose datasets from hematological neoplasms that occur specifically in either early life (JMML; Louka et al., 2021) or in adulthood (primary MF; Psaila et al., 2020). We integrated the 57,489 $Lin^{-}CD34^{+}$ cells sampled from tissues across normal human ontogeny with 19,524 $Lin^{-}CD34^{+}$ cells from JMML patients (n = 2) and 19,524 $Lin^{-}CD34^{+}$ cells from MF patients (n = 15). The total dataset of 96,537 cells was then interrogated for lineage specification as for Figure 2A. Compared to its normal counterpart (PBM), JMML HSPC had fewer lymphoid, erythroid, and megakaryocytic progenitors (Figure 6A), with the majority of the HSPC being defined as HSC/MPP or myeloid progenitors (Figure 6B). This is in keeping with JMML being a myeloid neoplasm that originates in the earliest HSC compartment with an expansion of an abnormal $CD38^{-}CD90^{+}$ primitive HSPC population; with a reduced or abnormal erythroid, megakaryocytic, and lymphoid output from JMML HSC *in vitro*; and a myeloid-biased reconstitution *in vivo* (Louka et al., 2021). When compared to normal ABM, MF HSPCs showed a dramatic expansion of megakaryocyte progenitors with an almost complete absence of lymphoid progenitors (Figures 6A and 6B), as previously reported (Psaila et al., 2020).

The developmentally regulated molecular features of the progenitors in which blood cancers originate may drive the distinct biology of the disease at different ages. To understand whether the abnormal HSPC compartments in JMML and MF share features with normal counterparts from a particular developmental stage, we compared HSC/MPP and lineage-specific progenitors from disease states to their counterparts from all tissue types through ontogeny. Hierarchical clustering of the number of differential genes revealed that HSC/MPP, myeloid, and erythroid progenitors in childhood JMML clustered with fetal counterparts rather than postnatal PBM, whereas MF HSPC clustered with postnatal counterparts (Figure 6C).

Differential gene expression analysis was performed to compare cells from JMML and MF to normal ontogeny in each lineage (Table S7). JMML HSC/MPP showed a higher expression of stem cell genes (e.g., *HOPX*, *SPINK2*, *CLEC9A*) compared to normal counterparts, and this ''stemness'' signature was retained in JMML myeloid progenitors when compared to normal counterparts (Figure 6D). Concomitantly, more mature myeloid genes such as *ELANE* and *CEBPD* were downregulated in the JMML myeloid progenitors compared to normal counterparts, suggesting a block in differentiation along the granulocytic/neutrophil lineage. Gene regulatory network analysis showed similarities of JMML HSC/MPP regulons with fetal HSC/MPP (FLI1, MEF2C, MECOM, and GATA2), which were also enriched in JMML myeloid progenitors (Figure 6E).

MF HSC/MPP showed a high expression of immune and inflammatory pathways (*HLA* genes, *GBP4*, and *IFITM1*), a matrix- and collagen-degrading enzyme (*MMP2*), and genes with oncogenic function (*MYCN*, *KRT8*, and *KRT18*) that have been correlated with cancer progression and poor survival (Fortier et al., 2013; Lai et al., 2017). Megakaryocyte progenitors from MF showed markedly increased expression of a subset of genes involved in cell-matrix interactions and cell adhesion (*CD9* and *MPIG6B)* when compared to the megakaryocyte progenitors from healthy control tissues. Similar to MF HSC/MPP, megakaryocyte progenitors in MF also showed an increased expression of oncogenes (*RAB6B*), interferon-inducible genes (*IFITM3*), and the cell-cycle regulator *CDK2AP1* (Figure 6F). GRN analysis highlighted the activation of inflammatory signaling pathways (IRF9, IRF1, ELF1, and STAT1 regulons) and cell cycle (CUX1) in MF HSC/MPP and megakaryocyte progenitors, as well as the resurgence of megakaryocyte-associated regulons (MEF2C, FLI1) that were most prominent in eFL HSC/MPP in the normal ontogeny dataset (Figures 5I and 6G).

DISCUSSION

Single-cell approaches have been extensively applied to understand normal and perturbed hematopoiesis and to define human prenatal blood and immune cells (Park et al., 2020; Popescu et al., 2019; Ranzoni et al., 2021). These studies, together with other observations, have highlighted the predominance of erythroid lineage cells in human FL (Popescu et al., 2019) and B lymphoid cells in FBM (O'Byrne et al., 2019). Differences in the proliferative capacity of fetal and postnatal HSPC have been described using functional studies (Bowie et al., 2006; Copley et al., 2013; Lansdorp et al., 1993; Muench et al., 1994). A switch from multipotent to largely oligo/unipotent stem cells has also been documented between fetal and adult life (Notta et al., 2016). However, HSPCs sampled from multiple time points over human ontogeny from first trimester to adulthood at single-cell resolution have not previously been reported.

In this study, we sought to define how hematopoiesis evolves during the human lifespan by interrogating a comprehensive transcriptomic dataset of HSPCs sampled from five hematopoietic tissues over four stages of ontogeny. Our data clearly demonstrate the changing frequencies of lineage-specific subsets that exist within the HSPC compartment, and suggest that the molecular programs that drive these changes are likely to originate in the HSC compartment. Notably, directly comparing

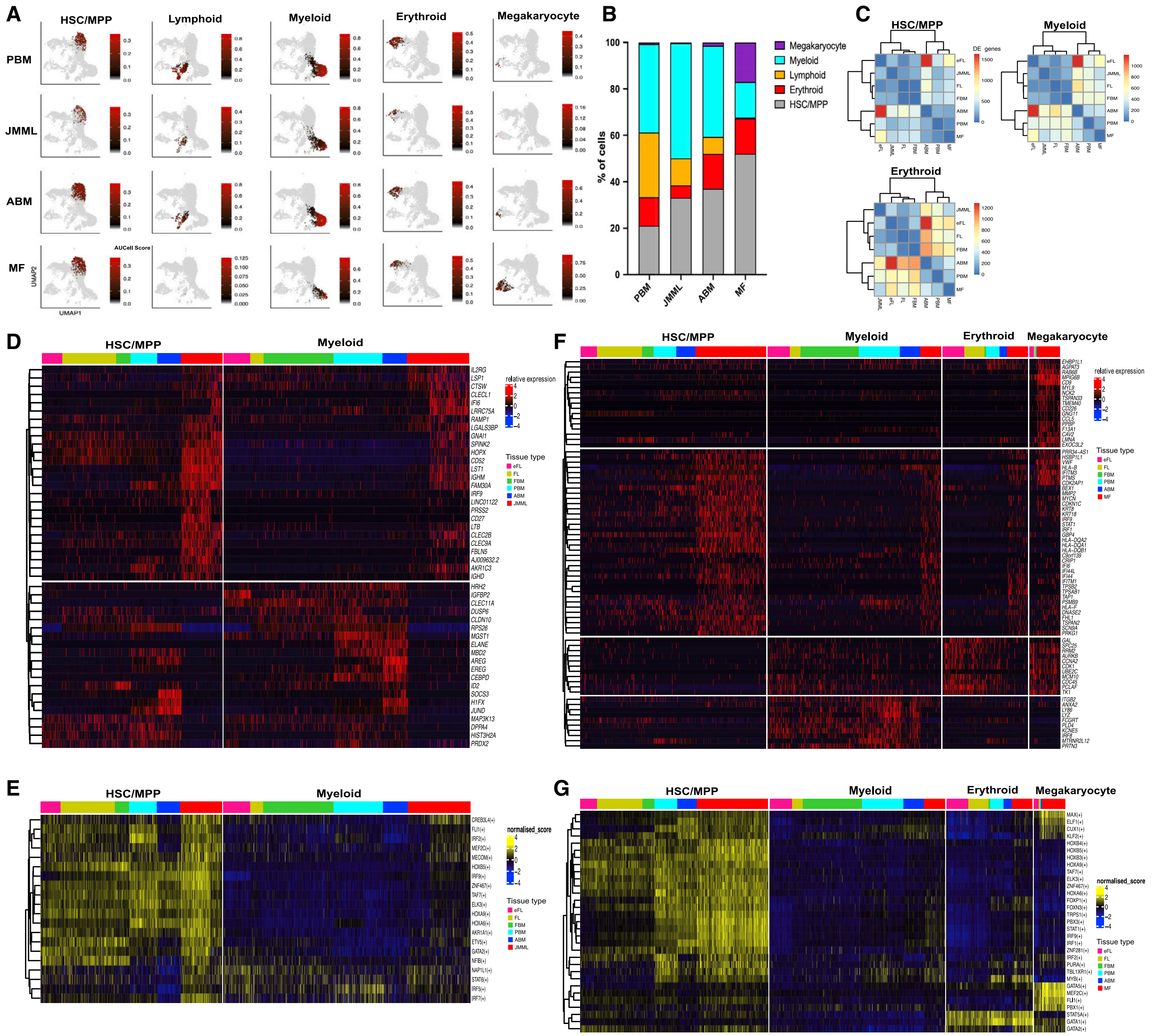

Figure 6. Concordance in gene expression between juvenile myelomonocytic leukemia (JMML) HSPCs and normal fetal HSPCs, and increased inflammatory signaling seen in myelofibrosis (MF) and ABM in HSPCs

Data were derived from JMML (n = 2, total cells = 19,524) and MF (n = 15, total cells = 19,524) HSPCs.

(A) UMAP plots showing lineage progenitors as identified by AUCell score >0.15 for downsampled datasets to show 5,600 cells per tissue type.

(B) The proportion of HSPCs (from all of the cells within each tissue), classified as HSC/MPP or lineage progenitors in JMML, MF, and their normal counterparts.

(C) Hierarchical clustering of the number of differentially expressed genes (DEGs) from pairwise comparisons of tissues from normal ontogeny and JMML and MF for HSC/MPP, myeloid and erythroid compartments (adjusted $p < 0.05$, absolute log2FC > 1.5, and expressing cell frequency >0.3 per tissue).

(D and E) Top DEGs and regulons, respectively, between JMML HSPCs and those from normal human ontogeny tissues for HSC/MPP and myeloid progenitors.

(F and G) Comparison of top DEGs and regulons, respectively, between MF HSPCs and those from normal human tissues through ontogeny for HSC/MPP and myeloid, erythroid, and megakaryocyte lineage progenitors.

FL and FBM HSPCs isolated from the same second trimester fetuses showed that while more than half of HSPCs in second trimester FLs are HSC/MPP, >80% of HSPCs in the matched FBM are lineage primed, with dramatic expansion of myelo-lymphopoiesis in FBM. This enrichment of oligo/unipotent progenitors and switch in lineage output is likely to be driven by microenvironmental cues, as hematopoiesis migrates from FL to BM during the second trimester, although this could also be explained by selective engraftment and the subsequent expansion of lympho-myeloid progenitors in the BM microenvironment. We also found key differences in the gene regulatory pathways between tissues, supporting previous studies indicating that a

complex interplay between cell-intrinsic (Dykstra et al., 2011; Grover et al., 2016) and cell-extrinsic factors (Ho et al., 2019) alter hematopoiesis and lineage specification during postnatal life. A marked and progressive increase in HSC quiescence was evident during postnatal development. As the cell cycle may play a role in determining the fate of multipotent progenitors, at least for erythro-megakaryocytic lineage specification (Lu et al., 2018; Tusi et al., 2018), it is possible that this may also play an instructive role in the myeloid-biased hematopoiesis that is observed with increasing age.

Finally, mapping of two hematological malignancies onto normal hematopoiesis datasets allowed us to identify the similarities and differences in lineage specification and GRNs between these diseases and normal hematopoiesis through ontogeny. Such comparisons showed a persistence of fetal-like gene expression programs in the childhood disease JMML, supporting a fetal origin for this disease (Helsmoortel et al., 2016). Myelofibrosis, a cancer that typically presents in later adulthood, showed further exacerbation of inflammatory signaling pathways initiated in adult BM, together with altered cell-matrix interactions and activation of oncogenic programs, and resurgence of megakaryocyte-associated transcriptional signatures that were prominent in FL, but subsequently downregulated in later physiological development.

In conclusion, defining the key similarities and differences between hematopoietic tissues across normal human ontogeny has provided a key resource to study how the characteristics of the most primitive HSPCs and their lineage fates change through the human lifetime. These results pave the way for a better understanding of hematopoiesis in normal human development, necessary to indicate the composition and likely long-term reconstitution ability of HSPCs selected from donors of different ages for cell-based therapies, including gene editing and stem cell transplantation (Harrison et al., 1997; Holyoake et al., 1999; Hua et al., 2019; Muench et al., 1994; Nicolini et al., 1999), as well as the cellular and molecular underpinnings of age-specific vulnerabilities to the origin and evolution of certain disease states.

STAR★METHODS

Detailed methods are provided in the online version of this paper and include the following:

- KEY RESOURCES TABLE
- RESOURCE AVAILABILITY
  - Lead contact
  - Materials availability
  - Data and code availability
- EXPERIMENTAL MODEL AND SUBJECT DETAILS
- METHOD DETAILS
  - Fluorescent activated cell sorting (FACS) staining, analysis and cell isolation
  - High-throughput single-cell RNA-sequencing (10x Chromium)
  - 10x Genomics single-cell RNA sequencing
  - Data processing and filtering of HSPC dataset
  - Data integration
  - Benchmarking distinct integrative methods for HSPC dataset
  - Data visualization with lineage signature gene sets
  - Clustering analysis, marker gene identification, and cell type annotation
  - Gene set enrichment analysis
  - Lineage progenitor quantification
  - Pseudotime trajectory analysis
  - Single-cell regulatory network inference and clustering (SCENIC) analysis
  - Mapping fetal liver cells to the reference dataset
  - Differential abundance analysis
- QUANTIFICATION AND STATISTICAL ANALYSIS

SUPPLEMENTAL INFORMATION

Supplemental information can be found online at https://doi.org/10.1016/j.celrep.2021.109698.

ACKNOWLEDGMENTS

We would like to acknowledge the contributions of the MRC WIMM Single Cell Facility and MRC-funded Oxford Consortium for Single-Cell Biology (MR/M00919X/1); WIMM Flow Cytometry Facility, which is supported by the MRC HIU, MRC MHU (MC_UU_12009); NIHR Oxford BRC; the Kay Kendall Leukaemia Fund (KKL1057); the John Fell Fund (131/030 and 101/517); the EPA fund (CF182 and CF170); and by the WIMM Strategic Alliance awards G0902418 and MC_UU_12025. We thank the High-Throughput Genomics Group at the Wellcome Trust Centre for Human Genetics (funded by Wellcome Trust Grant Reference 090532/Z/ 09/Z) and the NIH Intramural Sequencing Core and the National Institute of Health Research (NIHR) Oxford Biomedical Research Centre (BRC). The human fetal material was provided by the Joint MRC/Wellcome Trust grant 099175/Z/ 12/Z Human Developmental Biology Resource (https://hdbr.org). We also thank the donors who kindly provided samples for this research. A.R. was supported by a Bloodwise Clinician Scientist Fellowship (grants 14041 and 17001), Wellcome Trust Clinical Research Career Development Fellowship (216632/Z/19/Z), MRC Discovery award (MRCDA 0816-11), Lady Tata Memorial International Fellowship, and EHA-ASH Translational Research Training in Hematology Fellowship. B.P. was supported by a Wellcome Clinical Research Career Development Fellowship, a LAB282 award, an Oxford BRC Senior Research Fellowship, and a Cancer Research UK Advanced Clinician Scientist Fellowship. S.T. was supported by an Oxford-Bristol Myers Squibb (BMS) Fellowship.

AUTHOR CONTRIBUTIONS

A.R., D.I., S.O'B., N.E., J.O'S., G.B., and P.H. provided samples and/or performed experiments. G.W., E.F.H., W.X.W., and A.R.M. designed and performed the bioinformatic analyses. A.K., A.J.M., D.M.B., and I.R. contributed to the project oversight. A.R., B.P., and S.T. conceptualized the project, analyzed and interpreted the data, and wrote the manuscript. S.T. designed and developed the SingCellaR software. All of the authors read and approved the manuscript.

DECLARATION OF INTERESTS

The authors declare no competing interests.

Received: April 5, 2021
Revised: June 21, 2021
Accepted: August 19, 2021
Published: September 14, 2021

REFERENCES

Aibar, S., González-Blas, C.B., Moerman, T., Huynh-Thu, V.A., Imrichova, H., Hulselmans, G., Rambow, F., Marine, J.C., Geurts, P., Aerts, J., et al. (2017). SCENIC: single-cell regulatory network inference and clustering. Nat. Methods *14*, 1083–1086.

Bowie, M.B., McKnight, K.D., Kent, D.G., McCaffrey, L., Hoodless, P.A., and Eaves, C.J. (2006). Hematopoietic stem cells proliferate until after birth and show a reversible phase-specific engraftment defect. J. Clin. Invest. *116*, 2808–2816.

Büttner, M., Miao, Z., Wolf, F.A., Teichmann, S.A., and Theis, F.J. (2019). A test metric for assessing single-cell RNA-seq batch correction. Nat. Methods *16*, 43–49.

Copley, M.R., Babovic, S., Benz, C., Knapp, D.J., Beer, P.A., Kent, D.G., Wohrer, S., Treloar, D.Q., Day, C., Rowe, K., et al. (2013). The Lin28b-let-7-Hmga2 axis determines the higher self-renewal potential of fetal haematopoietic stem cells. Nat. Cell Biol. *15*, 916–925.

Dahlin, J.S., Hamey, F.K., Pijuan-Sala, B., Shepherd, M., Lau, W.W.Y., Nestorowa, S., Weinreb, C., Wolock, S., Hannah, R., Diamanti, E., et al. (2018). A single-cell hematopoietic landscape resolves 8 lineage trajectories and defects in Kit mutant mice. Blood *131*, e1–e11.

Drissen, R., Thongjuea, S., Theilgaard-Mönch, K., and Nerlov, C. (2019). Identification of two distinct pathways of human myelopoiesis. Sci. Immunol. *4*, eaau7148.

Dykstra, B., Olthof, S., Schreuder, J., Ritsema, M., and de Haan, G. (2011). Clonal analysis reveals multiple functional defects of aged murine hematopoietic stem cells. J. Exp. Med. *208*, 2691–2703.

Fortier, A.M., Asselin, E., and Cadrin, M. (2013). Keratin 8 and 18 loss in epithelial cancer cells increases collective cell migration and cisplatin sensitivity through claudin1 up-regulation. J. Biol. Chem. *288*, 11555–11571.

Giustacchini, A., Thongjuea, S., Barkas, N., Woll, P.S., Povinelli, B.J., Booth, C.A.G., Sopp, P., Norfo, R., Rodriguez-Meira, A., Ashley, N., et al. (2017). Single-cell transcriptomics uncovers distinct molecular signatures of stem cells in chronic myeloid leukemia. Nat. Med. *23*, 692–702.

Grover, A., Sanjuan-Pla, A., Thongjuea, S., Carrelha, J., Giustacchini, A., Gambardella, A., Macaulay, I., Mancini, E., Luis, T.C., Mead, A., et al. (2016). Single-cell RNA sequencing reveals molecular and functional platelet bias of aged haematopoietic stem cells. Nat. Commun. *7*, 11075.

Hao, Y., Hao, S., Andersen-Nissen, E., Mauck, W.M., 3rd, Zheng, S., Butler, A., Lee, M.J., Wilk, A.J., Darby, C., Zager, M., et al. (2021). Integrated analysis of multimodal single-cell data. Cell *184*, 3573–3587.e29.

Harrison, D.E., Zhong, R.K., Jordan, C.T., Lemischka, I.R., and Astle, C.M. (1997). Relative to adult marrow, fetal liver repopulates nearly five times more effectively long-term than short-term. Exp. Hematol. *25*, 293–297.

Hay, S.B., Ferchen, K., Chetal, K., Grimes, H.L., and Salomonis, N. (2018). The Human Cell Atlas bone marrow single-cell interactive web portal. Exp. Hematol. *68*, 51–61.

Helsmoortel, H.H., Bresolin, S., Lammens, T., Cavé, H., Noellke, P., Caye, A., Ghazavi, F., de Vries, A., Hasle, H., Labarque, V., et al. (2016). LIN28B overexpression defines a novel fetal-like subgroup of juvenile myelomonocytic leukemia. Blood *127*, 1163–1172.

Hie, B., Bryson, B., and Berger, B. (2019). Efficient integration of heterogeneous single-cell transcriptomes using Scanorama. Nat. Biotechnol. *37*, 685–691.

Ho, Y.H., Del Toro, R., Rivera-Torres, J., Rak, J., Korn, C., García-García, A., Macías, D., González-Gómez, C., Del Monte, A., Wittner, M., et al. (2019). Remodeling of Bone Marrow Hematopoietic Stem Cell Niches Promotes Myeloid Cell Expansion during Premature or Physiological Aging. Cell Stem Cell *25*, 407–418.e6.

Holyoake, T.L., Nicolini, F.E., and Eaves, C.J. (1999). Functional differences between transplantable human hematopoietic stem cells from fetal liver, cord blood, and adult marrow. Exp. Hematol. *27*, 1418–1427.

Hua, P., Roy, N., de la Fuente, J., Wang, G., Thongjuea, S., Clark, K., Roy, A., Psaila, B., Ashley, N., Harrington, Y., et al. (2019). Single-cell analysis of bone marrow-derived CD34+ cells from children with sickle cell disease and thalassemia. Blood *134*, 2111–2115.

Ivanovs, A., Rybtsov, S., Ng, E.S., Stanley, E.G., Elefanty, A.G., and Medvinsky, A. (2017). Human haematopoietic stem cell development: from the embryo to the dish. Development *144*, 2323–2337.

Kang, J.B., Nathan, A., Millard, N., Rumker, L., Moody, D.B., Korsunsky, I., and Raychaudhuri, S. (2020). Efficient and precise single-cell reference atlas mapping with Symphony. bioRxiv. https://doi.org/10.1101/2020.11.18.389189.

Kawabata, S., Ishita, Y., Ishikawa, Y., and Sakurai, H. (2015). Immediate-early response 5 (IER5) interacts with protein phosphatase 2A and regulates the phosphorylation of ribosomal protein S6 kinase and heat shock factor 1. FEBS Lett. *589*, 3679–3685.

Korotkevich, G., Sukhov, V., and Sergushichev, A. (2019). Fast gene set enrichment analysis. bioRxiv. https://doi.org/10.1101/060012.

Korotkevich, G., Sukhov, V., Budin, N., Shpak, B., Artyomov, M.N., and Sergushichev, A. (2021). Fast gene set enrichment analysis. bioRxiv. https://doi.org/10.1101/060012.

Korsunsky, I., Millard, N., Fan, J., Slowikowski, K., Zhang, F., Wei, K., Baglaenko, Y., Brenner, M., Loh, P.R., and Raychaudhuri, S. (2019). Fast, sensitive and accurate integration of single-cell data with Harmony. Nat. Methods *16*, 1289–1296.

Lai, Y.C., Cheng, C.C., Lai, Y.S., and Liu, Y.H. (2017). Cytokeratin 18-associated Histone 3 Modulation in Hepatocellular Carcinoma: A Mini Review. Cancer Genomics Proteomics *14*, 219–223.

Lansdorp, P.M., Dragowska, W., and Mayani, H. (1993). Ontogeny-related changes in proliferative potential of human hematopoietic cells. J. Exp. Med. *178*, 787–791.

Lavrovsky, Y., Chatterjee, B., Clark, R.A., and Roy, A.K. (2000). Role of redox-regulated transcription factors in inflammation, aging and age-related diseases. Exp. Gerontol. *35*, 521–532.

Leek, J.T., Johnson, W.E., Parker, H.S., Jaffe, A.E., and Storey, J.D. (2012). The sva package for removing batch effects and other unwanted variation in high-throughput experiments. Bioinformatics *28*, 882–883.

Louka, E., Povinelli, B., Rodriguez-Meira, A., Buck, G., Wen, W.X., Wang, G., Sousos, N., Ashley, N., Hamblin, A., Booth, C.A.G., et al. (2021). Heterogeneous disease-propagating stem cells in juvenile myelomonocytic leukemia. J. Exp. Med. *218*, e20180853.

Lu, Y.C., Sanada, C., Xavier-Ferrucio, J., Wang, L., Zhang, P.X., Grimes, H.L., Venkatasubramanian, M., Chetal, K., Aronow, B., Salomonis, N., and Krause, D.S. (2018). The Molecular Signature of Megakaryocyte-Erythroid Progenitors Reveals a Role for the Cell Cycle in Fate Specification. Cell Rep. *25*, 2083–2093.e4.

McWilliams, L., Su, K.Y., Liang, X., Liao, D., Floyd, S., Amos, J., Moody, M.A., Kelsoe, G., and Kuraoka, M. (2013). The human fetal lymphocyte lineage: identification by CD27 and LIN28B expression in B cell progenitors. J. Leukoc. Biol. *94*, 991–1001.

Muench, M.O., Cupp, J., Polakoff, J., and Roncarolo, M.G. (1994). Expression of CD33, CD38, and HLA-DR on CD34+ human fetal liver progenitors with a high proliferative potential. Blood *83*, 3170–3181.

Nicolini, F.E., Holyoake, T.L., Cashman, J.D., Chu, P.P., Lambie, K., and Eaves, C.J. (1999). Unique differentiation programs of human fetal liver stem cells shown both in vitro and in vivo in NOD/SCID mice. Blood *94*, 2686–2695.

Notta, F., Zandi, S., Takayama, N., Dobson, S., Gan, O.I., Wilson, G., Kaufmann, K.B., McLeod, J., Laurenti, E., Dunant, C.F., et al. (2016). Distinct routes of lineage development reshape the human blood hierarchy across ontogeny. Science *351*, aab2116.

O'Byrne, S., Elliott, N., Rice, S., Buck, G., Fordham, N., Garnett, C., Godfrey, L., Crump, N.T., Wright, G., Inglott, S., et al. (2019). Discovery of a CD10-negative B-progenitor in human fetal life identifies unique ontogeny-related developmental programs. Blood *134*, 1059–1071.

Park, J.E., Jardine, L., Gottgens, B., Teichmann, S.A., and Haniffa, M. (2020). Prenatal development of human immunity. Science *368*, 600–603.

Pellin, D., Loperfido, M., Baricordi, C., Wolock, S.L., Montepeloso, A., Weinberg, O.K., Biffi, A., Klein, A.M., and Biasco, L. (2019). A comprehensive single cell transcriptional landscape of human hematopoietic progenitors. Nat. Commun. *10*, 2395.

Popescu, D.M., Botting, R.A., Stephenson, E., Green, K., Webb, S., Jardine, L., Calderbank, E.F., Polanski, K., Goh, I., Efremova, M., et al. (2019). Decoding human fetal liver haematopoiesis. Nature *574*, 365–371.

Psaila, B., Wang, G., Rodriguez-Meira, A., Li, R., Heuston, E.F., Murphy, L., Yee, D., Hitchcock, I.S., Sousos, N., O'Sullivan, J., et al.; NIH Intramural Sequencing Center (2020). Single-Cell Analyses Reveal Megakaryocyte-Biased Hematopoiesis in Myelofibrosis and Identify Mutant Clone-Specific Targets. Mol. Cell *78*, 477–492.e8.

Ranzoni, A.M., Tangherloni, A., Berest, I., Riva, S.G., Myers, B., Strzelecka, P.M., Xu, J., Panada, E., Mohorianu, I., Zaugg, J.B., and Cvejic, A. (2021). Integrative Single-Cell RNA-Seq and ATAC-Seq Analysis of Human Developmental Hematopoiesis. Cell Stem Cell *28*, 472–487.e7.

R Core Development Team (2013). R: a language and environment for statistical computing (R Foundation for Statistical Computing).

Riquelme, P., Amodio, G., Macedo, C., Moreau, A., Obermajer, N., Brochhausen, C., Ahrens, N., Kekarainen, T., Fändrich, F., Cuturi, C., et al. (2017). DHRS9 Is a Stable Marker of Human Regulatory Macrophages. Transplantation *101*, 2731–2738.

Roy, A., Cowan, G., Mead, A.J., Filippi, S., Bohn, G., Chaidos, A., Tunstall, O., Chan, J.K., Choolani, M., Bennett, P., et al. (2012). Perturbation of fetal liver hematopoietic stem and progenitor cell development by trisomy 21. Proc. Natl. Acad. Sci. USA *109*, 17579–17584.

Schäfer, A., Mekker, B., Mallick, M., Vastolo, V., Karaulanov, E., Sebastian, D., von der Lippen, C., Epe, B., Downes, D.J., Scholz, C., and Niehrs, C. (2018). Impaired DNA demethylation of C/EBP sites causes premature aging. Genes Dev. *32*, 742–762.

Trapnell, C., Cacchiarelli, D., Grimsby, J., Pokharel, P., Li, S., Morse, M., Lennon, N.J., Livak, K.J., Mikkelsen, T.S., and Rinn, J.L. (2014). The dynamics and regulators of cell fate decisions are revealed by pseudotemporal ordering of single cells. Nat. Biotechnol. *32*, 381–386.

Tusi, B.K., Wolock, S.L., Weinreb, C., Hwang, Y., Hidalgo, D., Zilionis, R., Waisman, A., Huh, J.R., Klein, A.M., and Socolovsky, M. (2018). Population snapshots predict early haematopoietic and erythroid hierarchies. Nature *555*, 54–60.

Van de Sande, B., Flerin, C., Davie, K., De Waegeneer, M., Hulselmans, G., Aibar, S., Seurinck, R., Saelens, W., Cannoodt, R., Rouchon, Q., et al. (2020). A scalable SCENIC workflow for single-cell gene regulatory network analysis. Nat. Protoc. *15*, 2247–2276.

Velten, L., Haas, S.F., Raffel, S., Blaszkiewicz, S., Islam, S., Hennig, B.P., Hirche, C., Lutz, C., Buss, E.C., Nowak, D., et al. (2017). Human haematopoietic stem cell lineage commitment is a continuous process. Nat. Cell Biol. *19*, 271–281.

Welch, J.D., Kozareva, V., Ferreira, A., Vanderburg, C., Martin, C., and Macosko, E.Z. (2019). Single-Cell Multi-omic Integration Compares and Contrasts Features of Brain Cell Identity. Cell *177*, 1873–1887.e17.

Yuan, J., Nguyen, C.K., Liu, X., Kanellopoulou, C., and Muljo, S.A. (2012). Lin28b reprograms adult bone marrow hematopoietic progenitors to mediate fetal-like lymphopoiesis. Science *335*, 1195–1200.

Zhao, J., Jaffe, A., Li, H., Lindenbaum, O., Cheng, X., Flavell, R., and Kluger, Y. (2020). Detecting regions of differential abundance between scRNA-seq datasets. bioRxiv. https://doi.org/10.1101/711929.

Zheng, S., Papalexi, E., Butler, A., Stephenson, W., and Satija, R. (2018). Molecular transitions in early progenitors during human cord blood hematopoiesis. Mol. Syst. Biol. *14*, e8041.

STAR★METHODS

KEY RESOURCES TABLE

REAGENT or RESOURCE	SOURCE	IDENTIFIER
Antibodies		
Viability (ef506) dye	eBioscience	Cat# 65-0866-18
Lineage Cocktail (FITC)	eBioscience	Cat# 22-7776-72; RRID:AB_2043865
CD34 (PerCP-Cy5.5)	BioLegend	Cat# 343522; RRID:AB_2228973
CD38 (APC)	eBioscience	Cat# 17-0389-42; RRID:AB_1834353
CD45RA (PE)	eBioscience	Cat# 12-9459-42; RRID:AB_10718238
CD90 (BV421)	Biolegend	Cat# 328122; RRID:AB_2561420
CD123 (PE-Cy7)	eBioscience	Cat# 25-1239-42; RRID:AB_1257136
Biological samples		
human early fetal liver (1st trimester FL)	Human Developmental Biology Resource	1_eFL, 2_eFL
Human fetal liver (2nd trimester FL)	Human Developmental Biology Resource	1_FL, 2_FL
Human fetal bone marrow (matched to 2nd trimester FL)	Human Developmental Biology Resource	1_FBM, 2_FBM
Pediatric bone marrow	Imperial College NHS Trust	1_Paed_BM, 2_Paed_BM
Adult bone marrow	StemCell Technologies, Canada (cat no. 17001).	1_Adult_BM
Chemicals, peptides, and recombinant proteins		
Maxpar PBS Buffer	Fluidigm	Cat# 201058
10% BSA in Iscove's MDM	StemCell Technologies, Inc.	Cat# 09300
Ficoll-Paque PLUS	GE Healthcare Life Sciences	Cat#17-5442-03
CD34 MicroBead kit and MACS system	Miltenyi Biotech	Cat#130-046-703
CD34 MicroBead ultra	Miltenyi Biotech	Cat#130-100-453
RoboSep Buffer	StemCell Technologies	Cat# 20104
UltraPure EDTA	invitrogen	Cat# 15576-028
PBS pH 7.4	GIBCO (LifeTechnologies)	Cat# 10010-015
Fetal Bovine Serum (Heat inactivated)	Sigma	F7524
Critical commercial assays		
Chromium Single Cell 3′ GEM Library with Gel Bead Kit v3	10x Genomics, Inc.	Cat#1000092
Deposited data		
10X scRNaseq data in this manuscript	This manuscript	GSE155259
10X scRNaseq data in this manuscript	This manuscript	Mendeley Data (https://doi.org/10.17632/phfgms85x2.1)
10X scRNaseq data in this manuscript	(Louka et al., 2021)	GSE111895
10X scRNaseq data in this manuscript	(Psaila et al., 2020)	GSE144568
Software and algorithms		
R (v3.6.3, v.4.0.2, v.4.0.4)	(R Core Development Team, 2013)	https://cran.r-project.org/bin/macosx/
Flowjo version (10.5.3)	Flowjo	www.flowjo.com
RStudio (v1.1.463)	Team R Studio	https://www.rstudio.com/products/rstudio/download/
Cell Ranger v3.0.1	10x Genomics, Inc.	https://github.com/10XGenomics/cellranger

(Continued on next page)

CellPress
OPEN ACCESS

Continued

REAGENT or RESOURCE	SOURCE	IDENTIFIER
SingCellaR (v.1.1.1, v.1.2.0)	This manuscript	Github: https://github.com/supatt-lab/SingCellaR
		Zenodo: https://zenodo.org/record/5153387
monocle3_0.2.3.0	(Trapnell et al., 2014)	https://cole-trapnell-lab.github.io/monocle3/
pyscenic_0.10.4	(Van de Sande et al., 2020)	https://github.com/aertslab/pySCENIC
AUCell_1.12.0	(Aibar et al., 2017)	https://www.bioconductor.org/packages/release/bioc/vignettes/AUCell/inst/doc/AUCell.html
kBET_0.99.6	(Büttner et al., 2019)	https://github.com/theislab/kBET
lisi_1.0	(Korsunsky et al., 2019)	https://github.com/immunogenomics/LISI
Seurat_4.0.1	(Hao et al., 2021)	https://satijalab.org/seurat/
rliger_1.0.0	(Welch et al., 2019)	https://github.com/welch-lab/liger
Scanorama_1.7	(Hie et al., 2019)	https://github.com/brianhie/scanorama
sva_3.38.0	(Leek et al., 2012)	https://bioconductor.org/packages/release/bioc/html/sva.html
harmony_1.0	(Korsunsky et al., 2019)	https://github.com/immunogenomics/harmony
fgsea_1.18.0	(Korotkevich et al., 2021)	https://bioconductor.org/packages/release/bioc/html/fgsea.html
DAseq_1.0.0	(Zhao et al., 2020)	https://github.com/KlugerLab/DAseq
symphony_1.0	(Kang et al., 2020)	https://github.com/immunogenomics/symphony
**Other**		
BioRender	Graphical abstract created with BioRender.com.	https://biorender.com/

RESOURCE AVAILABILITY

Lead contact

Further information and requests for resources and reagents should be directed to and will be fulfilled by the lead contact, Supat Thongjuea (supat.thongjuea@imm.ox.ac.uk).

Materials availability

This study did not generate new unique reagents.

Data and code availability

- All raw and processed sequencing data generated in this study were deposited in the NCBI Gene Expression Omnibus (GEO: GSE155259) and Mendeley Data: https://doi.org/10.17632/phfgms85x2.1. Accession numbers and DOI are also listed in the Key resources table.
- SingCellaR open-source codes are available and maintained on GitHub and Zenodo listed in the Key resources table.
- Any additional information required to reanalyze the data reported in this paper is available from the lead contact upon request.

EXPERIMENTAL MODEL AND SUBJECT DETAILS

Donated fetal tissue (1st trimester FL and matched 2nd trimester FL and FBM) was provided by the Human Developmental Biology Resource (HDBR, https://www.hdbr.org). Fetal tissues were transported to the laboratory at 4°C and processed immediately as described previously (O'Byrne et al., 2019; Roy et al., 2012). Adult BM mononuclear cells were purchased from StemCell Technologies, Canada (cat no. 17001). Normal pediatric bone marrow was prospectively collected in accordance with the Declaration of Helsinki for sample collection and use in research. After filtering through a 70 micron cell strainer, samples were red cell and granulocyte depleted by density gradient separation using Ficoll-Paque PLUS (GE Healthcare Life Sciences, cat. no. 17-5442-03) and CD34

enrichment was carried out on freshly isolated mononuclear cells (MNC) from some of the samples using a Miltenyi CD34 MicroBead kit and MACS system (Miltenyi Biotech, cat. no. 130- 046-703). Developmental stage (age) and sex can be found in Table S1.

METHOD DETAILS

Fluorescent activated cell sorting (FACS) staining, analysis and cell isolation

Cells were stained with fluorophore-conjugated monoclonal antibodies (mAb; see Key resources table) in PBS with 2% FBS and 1mM EDTA for 30 minutes followed by two washes. FACS-sorting was performed using a Becton Dickinson Aria III or Fusion 2 as previously described (Psaila et al., 2020).

High-throughput single-cell RNA-sequencing (10x Chromium)

Cells were thawed, stained with FACS antibodies and sorted on a Becton Dickinson Aria III or Fusion 2 as described above and as per recommendations in the 10x Genomics single cell protocols. 15,000-24,000 Lin-CD34+ cells were FACS sorted from each sample, and processed as described in (Psaila et al., 2020). Samples were processed according to the 10x Genomics protocol using the Chromium Single Cell 3′ library and Gel Bead Kits v3 (10x Genomics). Cells and reagents were prepared and loaded onto the chip and into the Chromium Controller for droplet generation. RT was conducted in the droplets and cDNA recovered through demulsification and bead purification. Pre-amplified cDNA was used for library preparation, multiplexed and sequenced on a HiSeq 2500.

10x Genomics single-cell RNA sequencing

Demultiplexed FASTQ files were aligned to the human reference genome (GRCh38/hg38) using Cell Ranger software (version 3.0.1) from 10x Genomics. The Cell Ranger ''count'' standard pipeline was used to obtain the expression matrix of Unique Molecular Identifier (UMI) for each individual sample.

Data processing and filtering of HSPC dataset

We used SingCellaR (https://zenodo.org/record/5153387; https://github.com/supatt-lab/SingCellaR) to process each sample individually. The function 'load_matrices_from_cellranger' was used to read in data matrices from the Cell Ranger output. Cell and gene filtering was performed by assessing QC plots using the 'plot_cells_annotation' function. Cells meeting the following QC parameters were included in analyses (Table S1): UMI counts > 1,000 and ≤ maximum UMIs; number of detected genes > 500 and ≤ maximum number of detected genes; the percentage of mitochondrial gene expression ≤ limited percentage of mitochondrial gene expression (10% or 20% depending on an individual sample). Genes expressed in at least 10 cells were included. After filtering according to these criteria, 57,489 cells passed quality control (Table S1) and were included in downstream analyses.

Data integration

To perform data integration, the 'SingCellaR_int' R object was created. R object file names from individual samples were required as the input for the object. The function 'preprocess_integration' was performed to combine all of UMIs from all samples and cluster together with marker gene information into a single integrated R object. Using the function 'get_variable_genes_by_fitting_GLM_model' as previously described (Psaila et al., 2020), 949 highly variable genes, after removing ribosomal and mitochondrial genes, were identified and used for PCA analysis of the integrated dataset. Data normalization (using scaled UMI counts by normalizing each library size to 10,000 and transforming to the log scale) and dimensional reduction were performed using the function 'normalize_UMI' and 'runPCA', with the fast PCA analysis from the IRLBA package. To perform integration, the function 'runSupervised_Harmony' (implemented in SingCellaR) was run by using 40 principal components (PCs) as determined by the PCA elbow plot generated using the 'plot_PCA_Elbowplot' function.

runSupervised_Harmony function automatically selects marker genes derived from differentially expressed genes for all clusters in all samples, to build up a gene set database for GSEA analysis. A gene set name in the built database is assigned with a cluster id and a sample id. The pre-ranked differentially expressed genes for each cluster are used as the input for GSEA. To quantify the enrichment of gene sets for each cluster per sample, GSEA is then performed using the fgsea package. GSEA is sequentially performed, starting from the first cluster of the first sample through to the last cluster of the last sample, to determine the significance level and the positive enrichment scores for the whole gene sets for each cluster. The enrichment scores from GSEA for each cluster from all samples are then transformed to a matrix of 'cluster matching' scores. The matrix is used as the input for an automated hierarchical clustering with the 'cutree' function to identify similarity of clusters across samples. This is used to estimate the number of likely matched and unmatched clusters from all samples. Common and unique clusters across samples are then identified from the hierarchical clustering tree. A matrix is created containing the prior probability for each single cell belonging to the common and unique clusters. Finally, the probability matrix is used as the input 'cluster_prior' together with the 'donor' and 'batch' as the covariates for the subsequent Harmony analysis (Korsunsky et al., 2019).

Benchmarking distinct integrative methods for HSPC dataset

We performed kBET and LISI methods (Büttner et al., 2019; Korsunsky et al., 2019) on our HSPC dataset (Figure S1) to benchmark the SingCellaR integrative method against previously published methods for integrating datasets. To run kBET, we annotated clusters of

HSPCs using AUCell score (Aibar et al., 2017). We selected cells with a high AUCell score (> 0.15) of HSC/MPP, myeloid, lymphoid, erythroid, megakaryocyte, eosinophil/basophil/mast and endothelial progenitor cell gene signatures (Table S4) and classified them into 7 groups. Due to strong AUCell score, indicating strong expression of signature genes per each selected lineage progenitor, we would expect that each group of cells should be aggregated well together when applied integrative methods. We next performed each data integration method and UMAP analysis. UMAP 2D-coordinates for all methods were used as the input matrices for kBET analysis. kBET analysis was run for each group of annotated lineage progenitor cells by subsampling 1,000 cells/group and donor information was used as the batch of interest. kBET average acceptance rate per group of HSPCs was calculated and plotted for each integration method (Figure S1D). For LISI, the function "compute_lisi" was performed with the input UMAP 2D-coordinates together with donor and annotated lineage information. iLISI and cLISI scores were calculated and plotted for each integration method (Figure S1E).

Data visualization with lineage signature gene sets

After data integration, data embedding methods were performed for visualization using SingCellaR. These included UMAP, force-directed graph, and diffusion map using functions 'runUMAP', 'runFA2_ForceDirectedGraph', and 'runDiffusionMap'. We investigated the expression of lineage signature gene sets superimposed on top of those embeddings. Lineage signature gene sets were collated by curating known canonical lineage markers selected from multiple published hematopoiesis datasets (Table S4).

SingCellaR calculates a lineage gene score for each cell based on the average gene expression of each gene set. Represented colors for gene sets can be assigned automatically or by user-defined colors. Transparency factors for selected colors are calculated from normalized expression values across cell types. SingCellaR uses 'ggplot2′ functionality for adding the dynamic alpha parameter values to "geom_point" to control the transparency of colors.

Clustering analysis, marker gene identification, and cell type annotation

Clustering analysis was performed using the function 'identifyClusters' in SingCellaR with the integrative embeddings as the input together with the 'cosine' as a distant metric and local k-nearest neighbor (KNN) equal to 30. SingCellaR clusters cells using k-nearest neighbor approach implemented by a fast KNN algorithm from 'RcppAnnoy' package. After nearest neighbors are identified, the weighted graph is created with weight values calculated from normalized shared number of the nearest neighbors. The 'louvain' community detection method implemented by igraph package was applied to identify clusters. To identify genes differentially expressed in each cluster, the function 'findMarkerGenes' was performed. SingCellaR uses a standard nonparametric Wilcoxon test on log-transformed, normalized UMIs to compare expression level. Fisher's exact test was used to compare the expressing cell frequency of each gene as previously described (Giustacchini et al., 2017). *P*-values generated from both tests were then combined using Fisher's method and adjusted using the Benjamini-Hochberg (BH) correction. Genes expressed by each individual cluster are compared to all other clusters and differential genes defined as an absolute log2 fold change of $\geq$ 1.5 and adjusted *P*-value of < 0.05, with the fraction of expressing cell frequency of > 0.3. Differentially expressed genes were ranked using *P*-values and log2FC to select the top differential genes per cluster.

To annotate clusters, the function 'identifyGSEAPrerankedGenes' was used to pre-rank genes obtained from differential gene expression analysis comparing each individual cluster with all other clusters. Gene ranking scores were calculated as the log2 of expression fold-change multiplied by -log10 of the adjusted *P*-value. To run GSEA, the function 'Run_fGSEA_for_multiple_comparisons' was performed using the fgsea package (Korotkevich et al., 2019). The function 'plot_heatmap_for_fGSEA_all_clusters' was used to perform hierarchical clustering and to visualize GSEA positive enrichment scores from all clusters on the heatmap. This cell annotation heatmap (Figure 1C) together with the identified top marker genes (Table S2) and manual curation was used to identify cell clusters.

Gene set enrichment analysis

SingCellaR provides functions for GSEA analysis. These functions include: 'Run_fGSEA_analysis' used to compare two groups of cells; 'Run_fGSEA_for_a_selected_cluster_vs_the_rest_of_clusters' to compare any selected cluster against all other clusters; 'Run_fGSEA_for_multiple_comparisons' function to performing GSEA on multiple comparisons. Curated lineage signature gene sets used here are listed in Table S3. The HALLMARK gene set was downloaded from MSigDB (http://software.broadinstitute.org/gsea/msigdb/collections.jsp). Genes were pre-ranked using the function 'identifyGSEAPrerankedGenes'.

Lineage progenitor quantification

AUCell (Aibar et al., 2017) was used to quantify the number of cells affiliated to each lineage (HSC/MPP, myeloid, lymphoid, erythroid, megakaryocyte, and eosinophil/basophil/mast) among all HSPCs). To run AUCell, the function 'Build_AUCell_Rankings' was performed followed by 'Run_AUCell' with the gene matrix transposed (GMT) file of lineage gene sets. We manually inspected different AUCell thresholds per each lineage. We selected cells with AUCell score > 0.15 for each lineage and calculated the proportion of cells in each lineage progenitor subset and between tissues.

Pseudotime trajectory analysis

We used Monocle3 (version: 0.2.3.0) combining with the SingCellaR results to identify trajectories of the entire dataset. Raw UMI count data and clustering annotations were extracted from SingCellaR object to build a Monocle 'cds' object. We first used

'preprocess_cds' function to normalize the data. The UMAP and forced-directed graph dimensional reduction and clustering results slots were obtained from SingCellaR analyses. The trajectory was identified using "learn_graph" function on the UMAP reduction embeddings, representing paths along different lineages. "Order_cells" function was used to calculate pseudotime and the root node was defined using function "get_earliest_principal_node" on the UMAP reduction embeddings. The trajectory was defined from the graph plot as: 1) HSC – Ery: cl1-cl4-cl3-cl9; 2) HSC – Lym: cl1-cl12-cl6-cl10-cl8; 3) HSC – MKE: cl1-cl4-cl13; 4) HSC – Mye: cl1-cl7-cl5-cl2. Cluster 10 was confirmed as an intermediate proliferative state by repeating analyses after removal of cell cycle effect. We further downsampled the number of cells to control for differences in numbers of cells captured from each tissue (HSC – Ery: 1,650 cells per tissue; HSC – Lym: 1,648 cells per tissue; HSC – MKE: 1,363 cells per tissue; and HSC – Mye: 2,715 cells per tissue). Selected marker genes for each lineage were presented in heatmaps using the ComplexHeatmap package and line plot using ggplot2.

Single-cell regulatory network inference and clustering (SCENIC) analysis

We used pyscenic (version 0.10.4) to perform single-cell regulatory network analysis. We performed the analysis by following the protocol steps described in SCENIC workflow (Van de Sande et al., 2020). We analyzed the cells corresponding to HSC/MPP and main lineage progenitor sub-types described as the AUCell score of > 0.15 (Figure 2A). We first run the python script 'arboreto_with_multiprocessing.py' using the 'grnboost2' method followed by running the 'pyscenic' using default parameters with the database file 'hg38__refseq-r80__10kb_up_and_down_tss.mc9nr.feather' and the motif information file 'motifs-v9-nr.hgnc-m0.001-o0.0.tbl'. The AUCell analysis was further performed using 'pyscenic aucell' function with parameters 'rank_threshold' 5000, 'auc_threshold' 0.05 and 'nes_threshold' 3. Identified regulons from pyscenic were further selected based on the average AUCell score across cells > 0.02 and the number of genes in each regulon > 10. Differential regulons were selected using the visual inspection on the heatmap plot of normalized AUCell scores across tissues or lineages.

Mapping fetal liver cells to the reference dataset

We applied Symphony (Kang et al., 2020) to map fetal liver cells from our dataset to the reference dataset (Popescu et al., 2019). For mapping, we used the pre-built reference 'atlas of human fetal liver hematopoiesis' provided by Symphony package. The 'mapQuery' function was performed using the gene expression data and cell metadata from our dataset. We further applied the 'knnPredict' (k = 30) function to transfer the cell types from the reference dataset to the clusters identified in our data. We plotted cells from our fetal liver dataset overlaid on top of the reference dataset (Figure S2D).

Differential abundance analysis

DA-Seq (Zhao et al., 2020) analysis for pairwise comparisons of tissues across ontogeny was performed using 40 dimensions from PCA analysis, cell metadata, and UMAP derived from the SingCellaR object as the input. The parameter 'k.vector' was equal to 30-500 with steps of 30. We set the threshold for logistic classifier prediction at the absolute value of 0.85 to indicate differentially abundant cells.

QUANTIFICATION AND STATISTICAL ANALYSIS

All statistical analyses were performed in R statistical computing software. SingCellaR package was used to perform the differential gene expression analysis shown in Tables S2, S5, S6, and S7, and Figure S4. For the comparison of single-cell expression levels, a nonparametric Wilcoxon test was used, and Fisher's exact test was used to compare expression frequencies between defined populations. *P*-values generated from both tests were combined using Fisher's method and adjusted using the Benjamini-Hochberg (BH) correction. AUCell scores in different lineages across tissues (Figures 5F, 5G, and S5C) were compared using a Wilcoxon test and how significance was defined can be found in the legends.

Cell Reports, Volume *36*

Supplemental information

Transitions in lineage specification and gene regulatory networks in hematopoietic stem/progenitor cells over human development

Anindita Roy, Guanlin Wang, Deena Iskander, Sorcha O'Byrne, Natalina Elliott, Jennifer O'Sullivan, Gemma Buck, Elisabeth F. Heuston, Wei Xiong Wen, Alba Rodriguez Meira, Peng Hua, Anastasios Karadimitris, Adam J. Mead, David M. Bodine, Irene Roberts, Bethan Psaila, and Supat Thongjuea

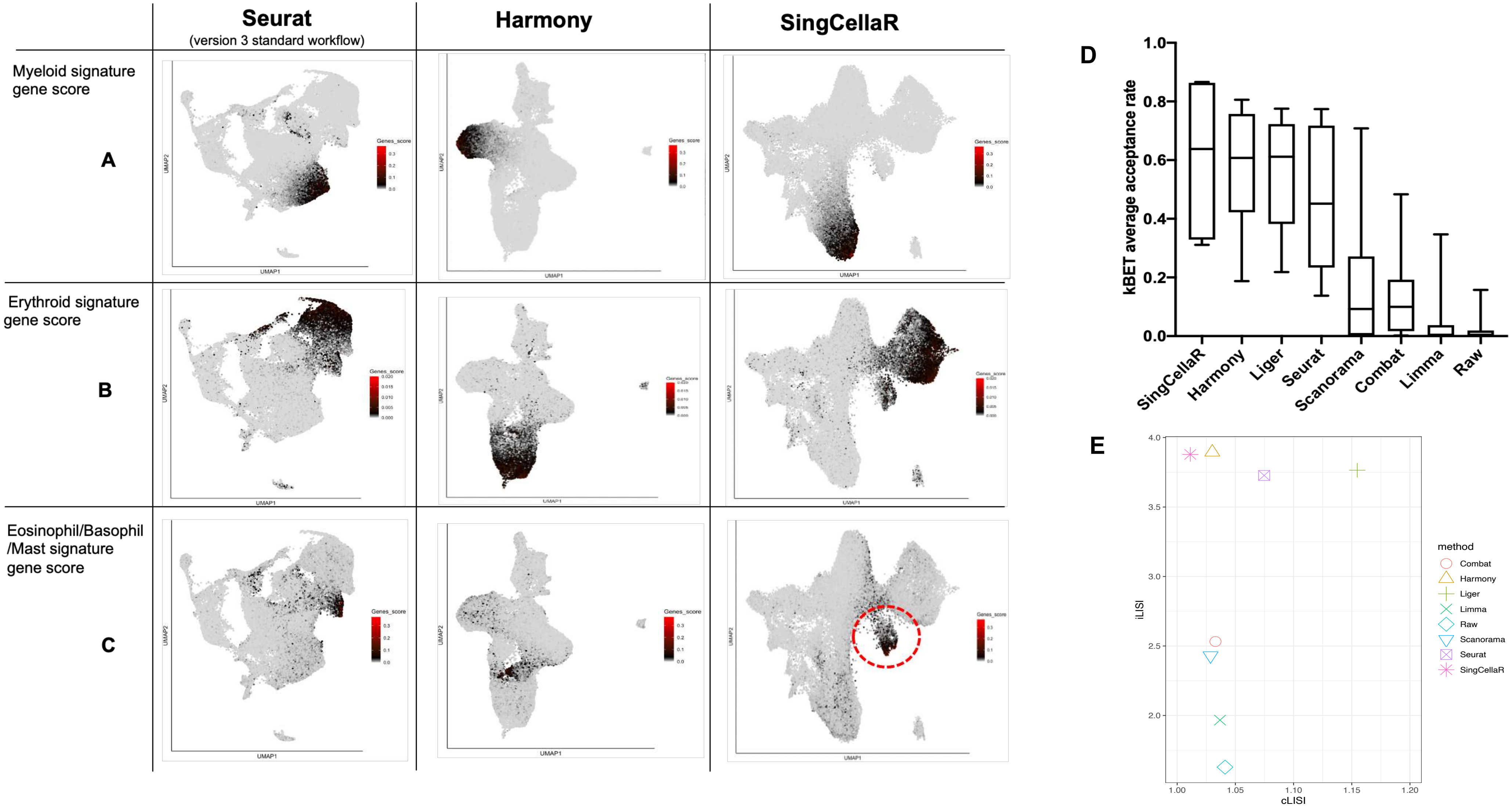

Supplemental Figure 1. Optimal resolution of lineage progenitor clusters was obtained using a 'supervised Harmony' integration approach, implemented in SingCellaR (related to Figure 1). UMAP plots of selected integration methods (Seurat, Harmony and SingCellaR) that performed well for this HSPC dataset. Lineage signature gene scores for (A) myeloid, (B), erythroid and (C) eosinophil/basophil/mast progenitors are superimposed on the UMAP plots, with a dashed circle highlighting the cluster of eosinophil/basophil/mast progenitor cells that is resolved only using SingCellaR. (D and E) Objective measures of integration for each method (D) Boxplot of kBET average acceptance rate score and (E) iLISI and cLISI scores. X-axis represents cLISI score. Y-axis represents iLISI score. A higher kBET (D) and iLISI score (E) indicates better data integration, and accurate integration should result in a higher iLISI score and cLISI score close to 1.

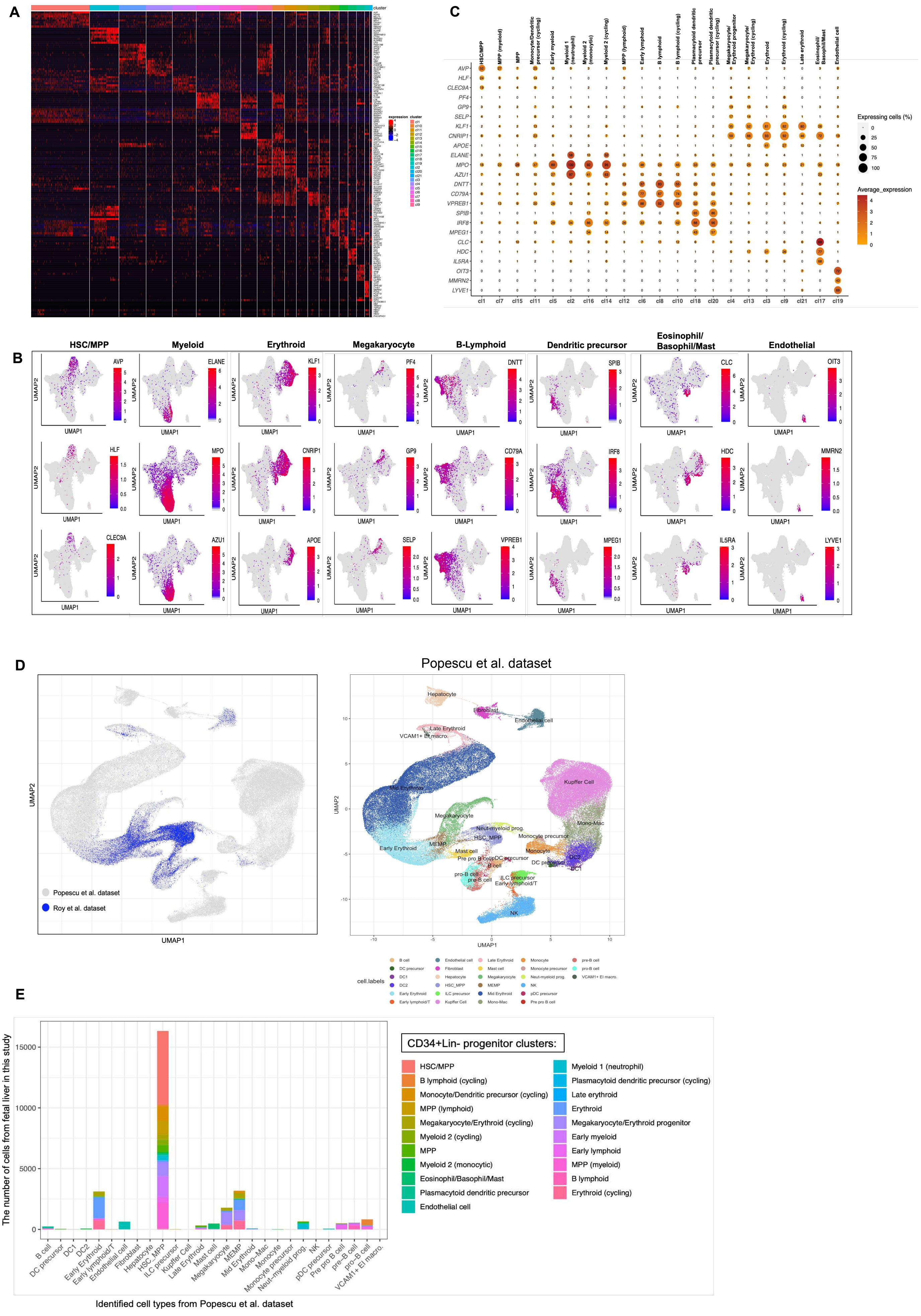

Supplemental Figure 2. Identification of cell clusters using differentially expressed genes (related to Figure 1). (A) Heatmap showing relative expression of the top 8 differentially expressed genes for each cluster. Cluster IDs are ranked from left (Cluster 1) to right (Cluster 21). (B) UMAP plots displaying the expression of canonical lineage marker genes. (C) Bubble plots showing the expression of lineage marker genes for each cluster. The size of the dot represents the percentage of cells expressing each gene (with the actual % shown inside the dot), and the colour represents the expression level. (D) Left panel – UMAP-plot displaying the mapping of fetal liver cells (from both 1st and 2nd trimester samples) from this study (Roy et al., blue dots) overlaid on top of the fetal liver reference dataset from Popescu et al. (grey dots) using the Symphony algorithm; Right panel – UMAP-plot displaying the annotated cell types in the Popescu et al. dataset. (E) Bar chart showing the mapping of fetal liver cells from this study on the annotated cell clusters from the Popescu et al. dataset.

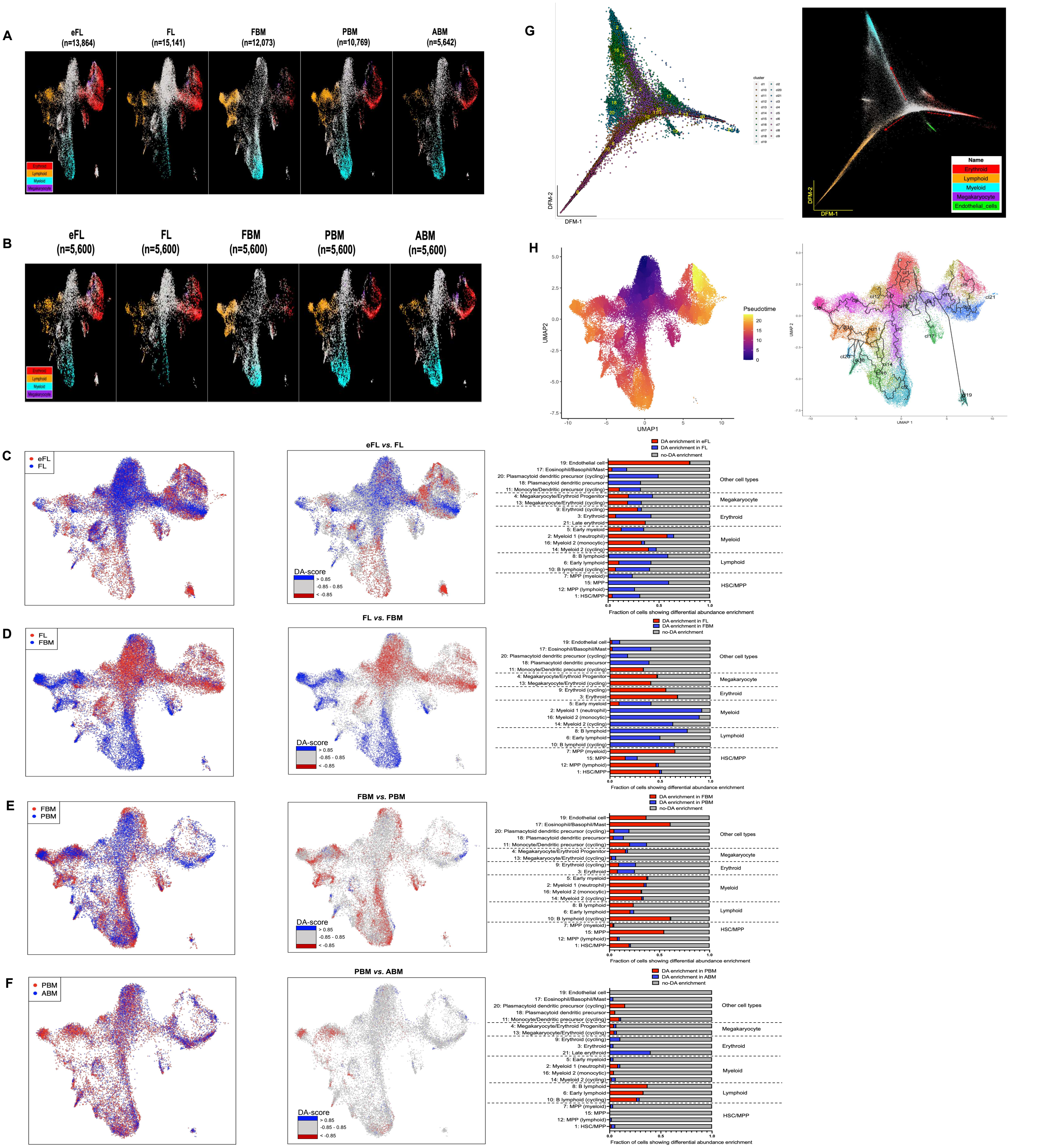

Supplemental Figure 3. Dynamic changes in the cellular composition of HSPCs across tissues and differentiation trajectory analyses (related to Figures 2 and 3). UMAP plots overlaid with lineage gene signature scores for HSC/MPP (grey); erythroid (red); lymphoid (yellow); myeloid (cyan) and megakaryocyte (purple) (A) showing all cells in each tissue (B) showing down-sampled cells (5600 cells per tissue). (C-F) Differential abundance (DA) analysis using DA-seq. Left panel, UMAP plots showing cells from pairwise comparisons of (C) eFL *vs.* FL, (D) FL *vs.* FBM, (E) FBM *vs.* PBM, and (F) PBM *vs.* ABM. Middle panel, UMAP plots showing the logistic classifier prediction for differentially abundant regions for each comparison. Cells in red are predicted to be more abundant in the first tissue with DA-score (using a cut-off score of < -0.85), whereas cells in blue indicate high abundance prediction in the second tissue (DA-score > 0.85). Cells in grey do not have a substantial DA score. Right panel, bar charts display the fraction of abundant cells per each cluster for each comparison. Red bar indicates the fraction of abundant cells per cluster in the first tissue. Blue bar indicates the fraction of cells in each cluster that have a high DA score, suggesting differential abundance in one tissue *vs.* the other. Grey bar indicates the fraction of cells in non-differentially abundant regions for each cluster. (G) HSPCs differentiation trajectories on a diffusion map. Left panel, diffusion map with cluster ID information. Right panel, diffusion map with the superimposition of 5 lineages progenitor gene sets. Red - erythroid; yellow - lymphoid; cyan - myeloid; purple - megakaryocyte; green - endothelial cells. The arrows indicate the main trajectories of cell differentiation from HSC/MPP towards lymphoid, erythroid/megakaryocyte, and myeloid differentiation. (H) Pseudotime and trajectory graph using Monocle3 analysis. Left panel, UMAP-plot superimposed with pseudotime scale. Dark-blue indicates the starting state and yellow or red indicates the terminal state of the cellular trajectories. Right panel, UMAP-plot with clusters and identified trajectory paths based on the monocle analysis.

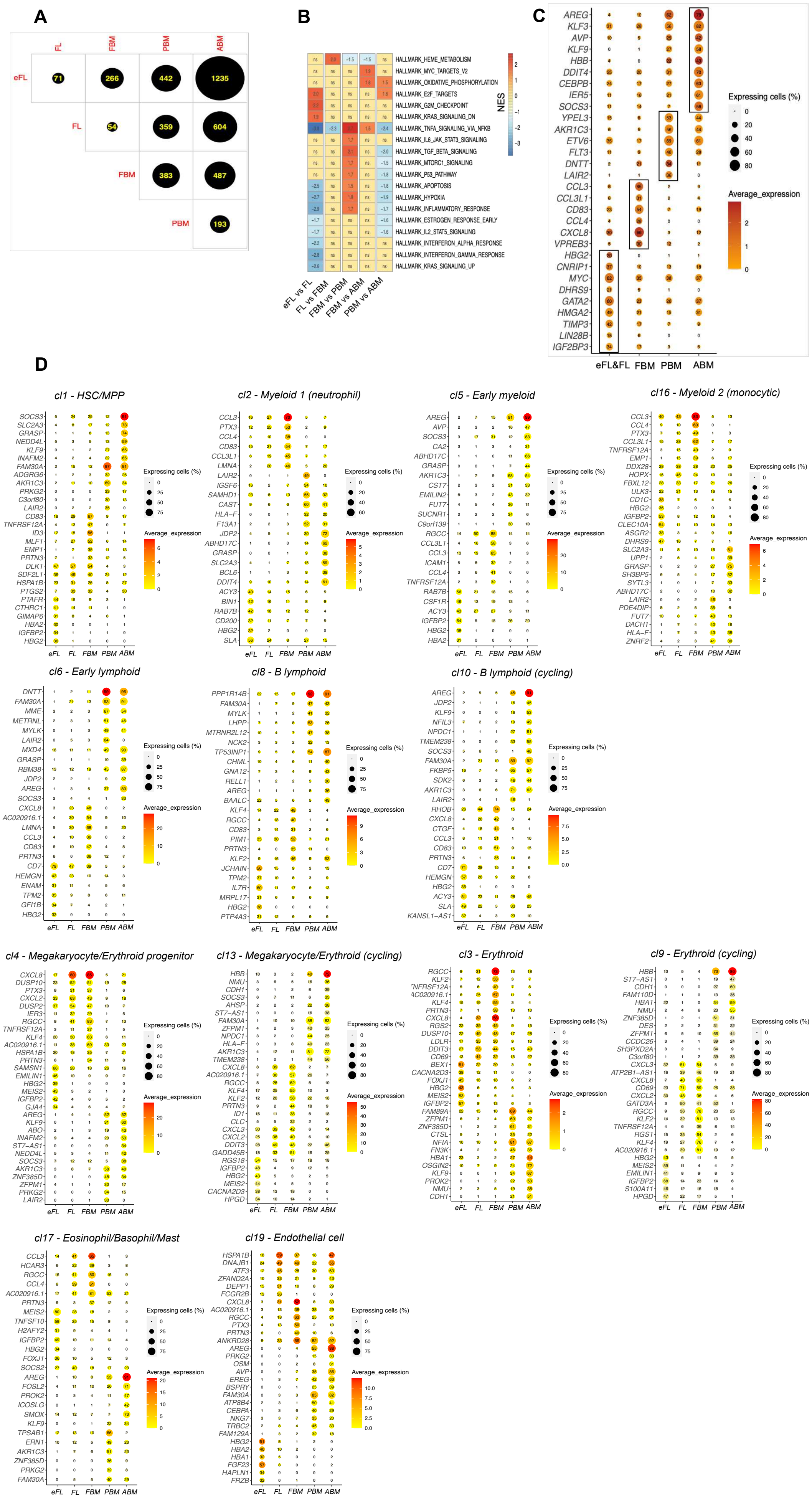

Supplemental Figure 4. Dynamic changes in HSPC gene expression programs across human ontogeny (related to Figure 2 and STAR Methods – Quantification and Statistical Analysis). (A) The number of differentially expressed genes between tissues in pairwise comparisons. (B) Heatmap of gene set enrichment scores for HALLMARK gene sets for the comparison of 5 selected pairs of tissues – eFL *vs.* FL, FL *vs.* FBM, FBM *vs.* PBM, FBM *vs.* ABM, and PBM *vs.* ABM. The gradient of colors represents the normalised enrichment score (NES) provided by GSEA. Red – gene sets enriched in the first tissue of the pair (positive values); blue – enrichment score for the second tissue of the pair (negative values). Yellow – no significant difference (‘ns’). (C) Selected tissue-specific genes that are highly expressed in each tissue. (D) The top 6 significantly upregulated genes for selected lineage clusters for each tissue compared to all other tissues.

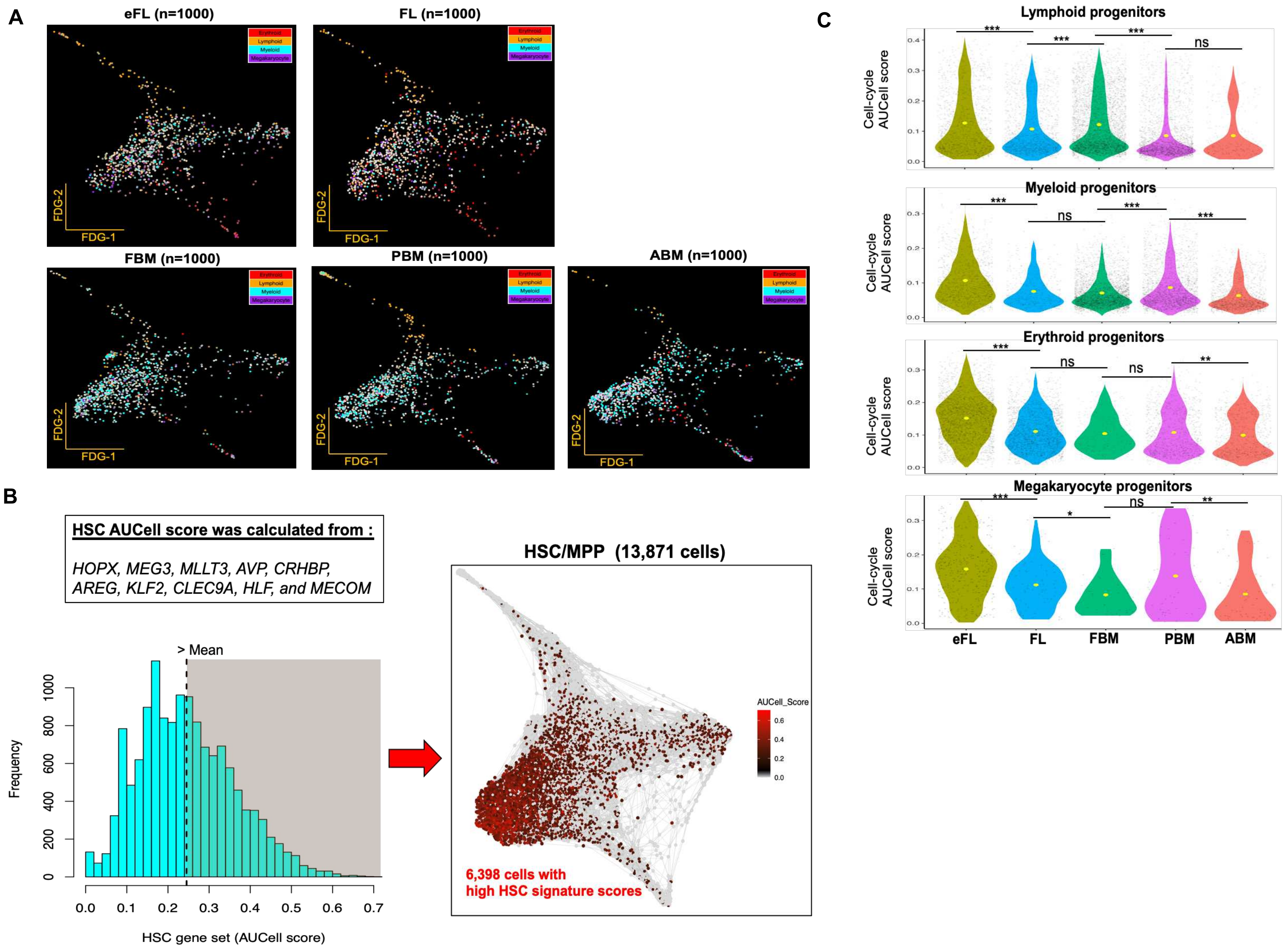

Supplemental Figure 5. Selection of primitive HSCs and comparison of AUCell scores for selected gene sets across tissues (related to Figure 5). (A) Force-directed graph trajectory analysis of selected HSC/MPPs across tissues following 'down-sampling' to 1,000 cells per tissue. Lineage signature gene expression is superimposed on top of the graph. Red - erythroid; yellow - lymphoid; cyan -myeloid; purple – megakaryocyte progenitors. (B) Left panel, the AUCell score distribution calculated from 10 curated HSC genes. 6,398 cells had higher HSC score than the average (>0.25) and were selected for downstream analyses. Right panel, force-directed graph represents selected HSC (black and red cells) in the trajectory of HSC/MPP compartment. (C) Violin plots comparing cell cycle AUCell scores in different lineages across tissues. Significance level shown as obtained using a Wilcoxon test (* = $P \leq 0.05$; ** = $P \leq 0.01$; *** = $P \leq 0.001$; and 'ns' = not significant).
